# Cone Beam CT Imaging-Guided Bronchoscopy vs CT Imaging-Guided Transthoracic Needle Biopsy for Peripheral Pulmonary Lesion Diagnosis

**DOI:** 10.1016/j.chest.2026.02.038

**Published:** 2026-03-25

**Authors:** Pranjal Patel, David E. Ost, Brian Shaller, Jiwoon Chang, Aishwarya Yenepalli, Hee Jae Choi, Dwayne Free, Cyra-Yoonsun Kang, Eric Sumner, Carrie Cao, Harmeet Bedi

**Affiliations:** a Division of Pulmonary, Allergy, and Critical Care Medicine, Stanford University, Stanford, CA; b Department of Respiratory Care, Stanford Health Care, Stanford, CA; c Department of Pulmonology and Critical Care Medicine, The Permanente Medical Group, Roseville, CA; d Department of Pulmonary and Critical Care Medicine, University of California Davis Medical Center, Davis, CA; e Department of Pulmonary Medicine, The University of Texas MD Anderson Cancer Center, Houston, TX; f Division of Pulmonary and Critical Care Medicine, University of North Carolina, Chapel Hill, NC

**Keywords:** cone beam CT imaging, diagnostic accuracy, diagnostic yield, general radiology, interventional pulmonology, interventional radiology, lung cancer, lung nodule, peripheral pulmonary lesion, pulmonary critical care, pulmonary nodule, robotic bronchoscopy, transthoracic needle aspiration, transthoracic needle biopsy

## Abstract

**Background:**

The identification of peripheral pulmonary lesions (PPLs) has increased significantly, either incidentally or through lung cancer screening, necessitating more biopsies to differentiate between malignant and benign causes. Cone beam CT imaging-guided bronchoscopic biopsy (CBCT-GB) and CT imaging-guided transthoracic needle biopsy (CT-TTNB) are used commonly for these biopsies, but their comparative diagnostic abilities have not been studied.

**Research Question:**

How does CBCT-GB compare with CT-TTNB for diagnosing PPLs?

**Study Design and Methods:**

This single-center retrospective comparative cohort study analyzed PPLs biopsied at an academic center via either CBCT-GB or CT-TTNB. The primary outcome was diagnostic accuracy at the 24-month follow-up, defined as the proportion of cases yielding a specific diagnosis (malignant or nonmalignant) or a nonspecific diagnosis that remained accurate through 24 months of clinical follow-up. Secondary outcomes included complication rates, procedure duration, radiation exposure, and the need for additional diagnostic procedures.

**Results:**

Of 895 patients analyzed, 340 of 375 patients (90.7%) in the CBCT-GB group and 440 of 475 patients (92.6%) in the CT-TTNB group had a diagnostic result (*P* = .301; OR, 0.979; 95% CI, 0.939-1.020). Complications occurred in 4.3% of patients undergoing CBCT-GB and 41.6% of patients undergoing CT-TTNB (*P* < .001). Pneumothorax rates were 1.8% for CBCT-GB and 31.4% for CT-TTNB (*P* < .001), and severe bleeding or cardiorespiratory failure occurred in 3.3% and 6.0% of patients, respectively (*P* < .001). Among patients meeting criteria for upfront invasive mediastinal staging, 86.5% of patients undergoing CBCT-GB received it at the time of PPL biopsy, compared with 14.0% of patients after biopsy in the CT-TTNB group (*P* < .001). Median effective radiation dose was 8.6 mSv in the robotic bronchoscopy CBCT-GB group and 7.5 mSv in the CT-TTNB group (*P* =.074).

**Interpretation:**

Our results show that CBCT-GB demonstrated a 24-month diagnostic accuracy comparable with that of CT-TTNB while offering improved safety and concurrent mediastinal lymph node staging. These data support CBCT-GB as the optimal initial procedure for PPL diagnosis.

FOR EDITORIAL COMMENT, SEE PAGE 310

**Take-Home Points**

**Research Question:** How does cone beam CT imaging-guided bronchoscopy (CBCT-GB) compare with CT imaging-guided transthoracic needle biopsy (CT-TTNB) for diagnosing peripheral pulmonary lesions (PPLs)?

**Results:** Twenty-four-month diagnostic accuracy was 90.7% for CBCT-GB and 92.5% for CT-TTNB (*P* = .301; OR, 0.979), and complications occurred in 4.3% and 41.6% of patients, respectively (*P* < .001).

**Interpretation:** Our results show CBCT-GB achieved 24-month diagnostic accuracy comparable with that of CT-TTNB while offering improved safety and concurrent nodal staging, supporting its use as the preferred initial procedure for PPL diagnosis in appropriately resourced centers.

The detection of peripheral pulmonary lesions (PPLs) on CT imaging has increased because of an increase in screen-detected and incidental pulmonary nodules.^1^^,^^2^ Although some PPLs can be monitored with serial imaging, many patients, especially those at intermediate to high risk of malignancy, require tissue sampling to guide clinical decision-making. Therefore, understanding the diagnostic accuracy and safety of biopsy methods is essential.

Two primary nonsurgical biopsy methods are used for PPL sampling: CT imaging-guided transthoracic needle biopsy (CT-TTNB) and bronchoscopic transbronchial biopsy. CT-TTNB, considered the gold standard, achieves a diagnostic accuracy (DA) of approximately 90% (range, 75%-97%), but carries higher complication rates, including pneumothorax (approximately 21%), chest tube placement (approximately 7%), and hemorrhage (approximately 3%).^3^^,^^4^ In contrast, bronchoscopy, without advanced imaging or a robotic bronchoscope, historically has demonstrated lower DA (range, 53%-71%), but fewer complications, with pneumothorax occurring in 1% to 2% of patients and rarely requiring chest tube placement (< 1%).^5^^,^^6^

Recent technological advances in bronchoscopy have improved DA to levels comparable with that of CT-TTNB. Techniques such as electromagnetic navigation (EMN) bronchoscopy, robotic-assisted bronchoscopy (RAB), and digital tomosynthesis-guided bronchoscopy achieve DA rates of 73% to 87%.^7^, ^8^, ^9^, ^10^, ^11^, ^12^ Among these, cone beam CT imaging-guided bronchoscopy (CBCT-GB) has emerged as a promising innovation. CBCT-GB uses real-time 3-dimensional intraprocedural imaging to confirm precise instrument localization and tool-in-lesion confirmation, achieving a DA of up to 94% with a comparable or superior safety profile as other bronchoscopic methods.^13^, ^14^, ^15^, ^16^, ^17^, ^18^

However, the PPL biopsy literature remains limited by noncomparative small studies with inconsistent DA definitions.^4^^,^^6^^,^^19^, ^20^, ^21^ Variability in reference standards, follow-up periods, and reporting granularity can lead to discrepancies of up to 22% in DA across studies,^22^ complicating comparisons between technologies. To date, only 1 study has compared any bronchoscopic method (EMN-guided bronchoscopy) with CT-TTNB,^23^ and none have compared CBCT-GB with CT-TTNB. Given CBCT-GB’s high DA, low complication rate, and the advantage of simultaneous lymph node staging, we hypothesize CBCT-GB may offer superior diagnostic usefulness and safety compared with CT-TTNB for PPL diagnosis. This study provides the first direct comparison, to our knowledge, of CBCT-GB and CT-TTNB for PPL diagnosis, with clear, granular reporting aligned with recent guidelines,^19^^,^^20^^,^^24^ offering a comprehensive basis for evaluating these biopsy methods and enabling meaningful comparisons with past and future studies.

## Study Design and Methods

This retrospective comparative cohort study analyzed all PPLs biopsied at Stanford Health Care using CBCT-GB or CT-TTNB from October 2, 2020, through October 14, 2022. The study was approved by the institutional review board of Stanford University (Identifier: 67140) with a waiver of consent on October 12, 2022, because all procedures constituted standard institutional care.

### Patient Selection

Adults ≥ 18 years of age undergoing nonoperative thoracic biopsy procedures at the institution during the study period were eligible. Patients undergoing CBCT-GB were identified from a prospective log, whereas patients undergoing CT-TTNB were identified through institutional imaging software. Exclusion criteria included visible endobronchial lesions, no focal PPL target, thoracic biopsies outside the pulmonary parenchyma, procedures canceled before the proceduralist’s portion, and incomplete procedural reports. After an RAB platform was introduced on September 1, 2021, CBCT-GB procedures used RAB. To mitigate learning curve effects, the first 10 patients undergoing RAB were excluded.

### CBCT-GB Procedure

All procedures were performed by board-certified interventional pulmonologists, often with a fellow. Standardized airway, anesthetic, and ventilatory protocols were used to minimize CT imaging-to-body divergence and atelectasis.^25^ Patients received total IV anesthesia, paralysis, orotracheal intubation (endotracheal tube size, 8.5-9.0 mm), recruitment with 4 30-second inspiratory holds at 30 cm H_2_O, tidal volume of 10 to 12 mL/kg ideal body weight, PEEP of 10 to 20 cm H_2_O, and Fio_2_ of ≤ 0.6.

Guidance used fixed cone beam CT (CBCT) imaging systems with augmented fluoroscopy (Siemens Artis-zee [Siemens] and Philips Azurion [Philips]) during inspiratory breath holds as needed. Non-RAB procedures used slim, ultraslim, or therapeutic bronchoscopes (BF-P190, BF-MP190, or BF-TH190, respectively; Olympus) with or without an extended working channel (Edge Catheter; Medtronic). RAB procedures used the EMN-based Monarch platform (Johnson & Johnson MedTech).

After intubation, the CBCT imaging system was iso-centered, and patients undergoing RAB underwent EMN registration. After proceduralists navigated to the target lesion, an initial CBCT imaging spin delineated the lesion for augmented fluoroscopy and assessed bronchoscope positioning. Subsequent spins verified biopsy tool placement and guided adjustments until achieving tool-in-lesion confirmation. Radial endobronchial ultrasound (EBUS) was not used because it was not standard of care at our institution. Tissue was obtained using a 21-gauge needle (PeriView FLEX; Olympus), a 2.0-mm forceps (Radial Jaw 4; Boston Scientific), or 1.1-mm or 1.7-mm cryoprobes (ERBECRYO 2; Erbe Elektromedizin GmbH). Convex EBUS-guided lymph node sampling was performed when indicated. Chest radiography after the procedure assessed pneumothorax before discharge. Complications were managed according to standard of care.

### CT-TTNB Procedure

Patients were positioned supine or prone and remained conscious, with local anesthesia administered before the biopsy. Moderate sedation was provided as needed. Biopsies were performed under fan-beam CT imaging guidance, and the sampling method (fine-needle aspiration or core biopsy), needle gauge, and number of passes were at the proceduralist’s discretion. Gel foam slurry was instilled routinely into the tract during withdrawal. Chest radiography after the procedure assessed pneumothorax before discharge. Complications were managed per standard of care.

### Data Collection and Outcomes

Data were managed securely using the Research Electronic Data Capture web-based Health Insurance Portability and Accountability Act-compliant database.^26^ Collected data included patient demographics, medical history, PPL characteristics, procedural details, follow-up imaging, and clinical outcomes, ensuring no duplicate demographic information for the 10 patients with multiple PPL biopsies in the same procedure.

Index pathologic features were classified against a prespecified reference standard as malignant, specific nonmalignant, or nonspecific per predefined diagnostic accuracy criteria (Table 1); contingency tables are provided to facilitate alternative DA calculations (e-Table 1). The prespecified primary outcome was 24-month DA, excluding those without 24-month imaging (Table 1). An index pathologic result was diagnostic if it revealed a specific malignant or specific nonmalignant diagnosis; nonspecific findings were considered diagnostic if subsequent testing yielded a specific nonmalignant diagnosis or the lesion was stable or had shrunk at 24 months. Atypical cells or normal lung tissue were always nondiagnostic.

**Table 1:** Definitions for Classifying Biopsy Results and Outcomes

Index Pathologic Results	Reference Standard of Truth Result
Specific malignant: Malignant diagnosis confirmed on index pathologic analysis	Malignant: Malignant diagnosis confirmed on index or subsequent pathologic analysis
Specific nonmalignant: • Positive microbiological growth on index pathologic analysis • Index pathologic analysis demonstrating any of the following: myxoid cartilaginous tissue, hamartoma, aspirated foreign material, sclerosing pneumocytoma, lymphoid hyperplasia, pulmonary infarct, pulmonary myxoma, interstitial lung disease, amyloidosis, granulomatosis with polyangiitis, antiglomerular basement membrane disease	Nonmalignant specific diagnoses: • Positive microbiological growth on index or subsequent pathologic analysis • Index or subsequent pathologic findings demonstrating any of the following: myxoid cartilaginous tissue, hamartoma, aspirated foreign material, sclerosing pneumocytoma, lymphoid hyperplasia, pulmonary infarct, pulmonary myxoma, interstitial lung disease, amyloidosis, granulomatosis with polyangiitis, antiglomerular basement membrane disease • Granulomatous inflammation on index pathologic analysis managed as clinical sarcoid by treating physicians
Nonspecific: • Index pathologic analysis demonstrating any of the following: organizing pneumonia, fibroelastotic scar, granulomatous inflammation, atypical cells, necrosis, nonspecific inflammation, or nonspecific normal lung tissue	Benign based on clinical and imaging follow-up: • PPL without size change after 24 mo of follow-up imaging • PPL smaller or resolved on follow-up imaging
	Enlarging but indeterminate at 24 mo: • PPL enlarged on follow-up imaging and followed up for 24 mo, but without a diagnosis
	Lost to follow-up: • Patients without 24 mo of follow-up imaging
Primary outcome	
Diagnostic accuracy at 24 mo: The proportion of patients yielding a diagnostic result that remains accurate through 24 mo of clinical follow-up, divided by the total number of patients who achieved 24 mo of clinical follow-up (excluding indeterminate enlarging lesions and those lost to follow-up) Biopsies meeting the following criteria were considered diagnostic: 1. Specific malignant on index pathologic analysis 2. Specific nonmalignant on index pathologic analysis 3. Nonspecific on index pathologic analysis (excluding atypical cells and normal lung tissue) where PPL was found to have a nonmalignant specific diagnosis on follow-up 4. Nonspecific on index pathologic analysis (excluding atypical cells and normal lung tissue) where PPL determined to be benign based on 24-mo clinical imaging follow-up (remained stable or reduced in size on follow-up)	
Secondary outcomes	
Diagnostic accuracy at 12 mo: 1. The proportion of patients yielding a diagnostic result that remains accurate through 12 mo of clinical follow-up, divided by the total number of patients who achieved 12 mo of clinical follow-up (excluding indeterminate enlarging lesions and those lost to follow-up) 2. Diagnostic yield per ATS and CHEST Delphi statement: the proportion of patients yielding a specific malignant, specific nonmalignant, granulomatous inflammation, or organizing pneumonia diagnosis on index pathologic analysis, divided by the total number of patients with no regard to clinical follow-up data 3. Effect of PPL characteristics on diagnostic accuracy 4. Complication rates 5. Procedure duration 6. Radiation exposure 7. Need for additional diagnostic procedures 8. Subset comparisons of non-RAB and RAB CBCT-GB with CT-TTNB 9. Subset comparisons of procedures with and without simultaneous EBUS lymph node staging	

ATS = American Thoracic Society; CBCT-GB = cone beam CT imaging-guided bronchoscopy; CHEST = American College of Chest Physicians; CT-TTNB = CT imaging-guided transthoracic needle biopsy; EBUS = endobronchial ultrasound; PPL = peripheral pulmonary lesion; RAB = robotic-assisted bronchoscopy.

Secondary outcomes included 12-month DA and diagnostic yield (DY) defined per the American Thoracic Society and American College of Chest Physicians (CHEST) guidelines (added after guideline publication).^27^ Other outcomes included the effects of PPL characteristics on DA, complications, procedure duration, radiation exposure, and need for additional diagnostic procedures. Radiation exposure, measured as dose area product for CBCT-GB and dose length product for CT-TTNB, was converted to effective dose using factors of 0.16 mSv/Gy **×**cm^2^ and 0.014 mSv/mGy, respectively, to facilitate comparison.^28^, ^29^, ^30^, ^31^, ^32^ Subset analyses compared non-RAB vs RAB CBCT-GB with CT-TTNB and procedures with vs without EBUS lymph node staging (e-Appendix 1).

### Statistical Analysis

**Figure S2:**
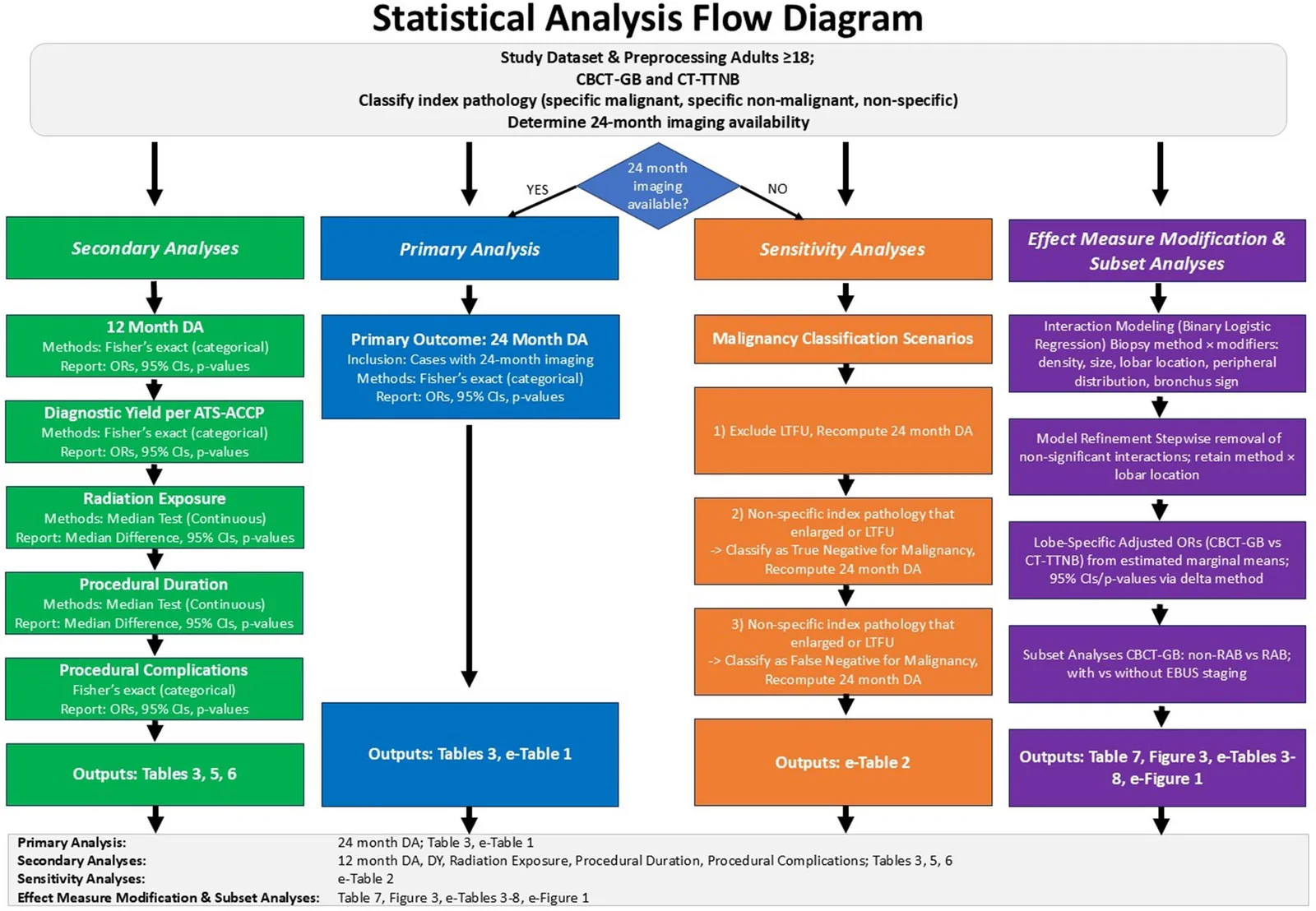


Descriptive statistics were used. Shapiro-Wilk testing indicated nonnormal distributions. Categorical variables are reported as counts and percentages with 95% CIs and were compared by Fisher's exact test (Bonferroni correction for higher-order subanalyses). Continuous variables are presented as medians with interquartile ranges and were compared using the Mann-Whitney *U* test or median test. Likelihood ratios were calculated for specific results (e-Fig 2). Three sensitivity analyses addressed malignancy classification for 24-month DA: (1) excluding patients lost to follow-up, (2) treating nonspecific index pathologic features in lesions that later enlarged or were lost to follow-up as true-negative findings for malignancy, and (3) treating them as false-negative findings.

Binary logistic regression modeling with biopsy method by modifier interaction terms assessed prespecified potential effect modifiers (PPL density, size, lobar location, peripheral distribution, and bronchus sign) for the primary outcome. A full interaction model was fit, then nonsignificant interactions were removed stepwise; the final model retained only the biopsy method by lobar location interaction.^33^^,^^34^ Adjusted lobe-specific ORs for CBCT-GB vs CT-TTNB were derived from estimated marginal means; 95% CIs and *P* values used the delta method. Sensitivity checks were performed using parameter estimates and the model-based covariance matrix. This approach was selected over propensity score matching, which would limit multivariable insight, would reduce sample size, would diminish generalizability, and would preclude assessing for effect measure modification.^35^^,^^36^

Statistical significance was set at a 2-tailed *P* value of < .05. Analyses used SPSS version 29.0.2.0 software (IBM) and adhered to the Standards for Reporting Diagnostic Accuracy Studies and Reporting Standards for Diagnostic Testing guidelines.^19^^,^^24^ This was neither an equivalence nor noninferiority analysis.

## Results

**Figure 1:**
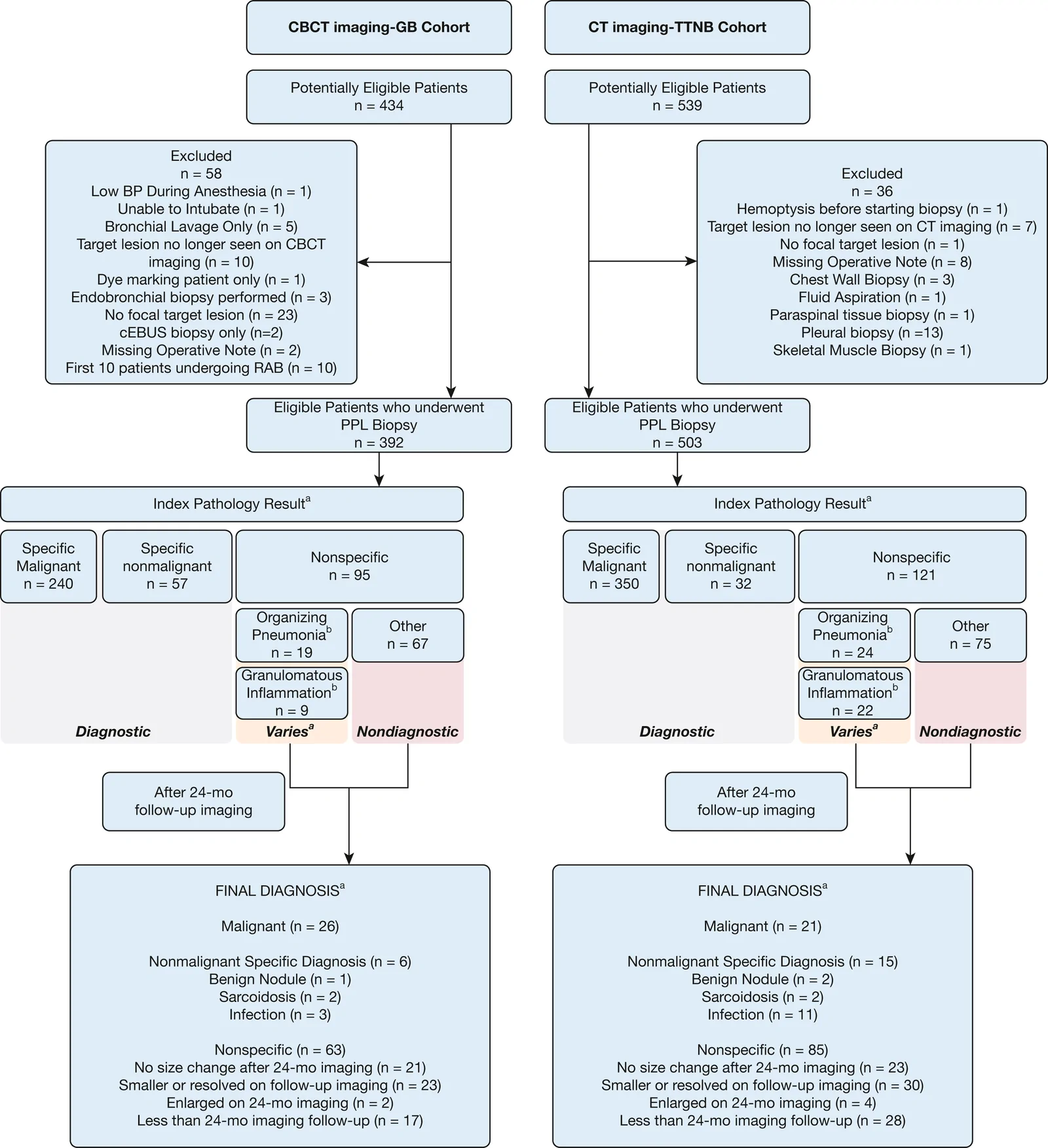
Flow diagram showing selection criteria, index pathologic results, and final diagnosis after 24-mo follow-up data. ^a^See Table 1 for definitions used for classification of index pathologic and final diagnosis results, and see e-Table 1 for precise counts within each bucket assignment. ^b^Organizing pneumonia and granulomatous inflammation are considered nondiagnostic for diagnostic accuracy calculations used in this study. However, they are considered diagnostic for diagnostic yield calculations (per American Thoracic Society and CHEST guidelines). CBCT = cone beam CT; CBCT-GB = cone beam CT imaging-guided bronchoscopy; cEBUS = convex endobronchial ultrasound; CHEST = American College of Chest Physicians; CT-TTNB = CT imaging-guided transthoracic needle biopsy; PPL = peripheral pulmonary lesion; RAB = robotic-assisted bronchoscopy.

Nine hundred seventy-three patients were screened during the study period. After excluding ineligible patients, 895 patients underwent a biopsy procedure: 392 patients were in the CBCT-GB group and 503 patients were in the CT-TTNB group (Fig 1). The CBCT-GB group included a higher proportion of White, non-Hispanic patients (55.2% vs 48.1%; *P* = .010), more patients with a current or former smoking history (55.5% vs 45.9%; *P* = .005), more patients without prior cancer history (42.8% vs 19.6%; *P* < .001), and fewer patients with a history of extrathoracic malignancy (40.8% vs 65.2%; *P* < .001). Other characteristics showed no significant differences (Table 2). PPL characteristics largely were similar, with the CBCT-GB group having more part-solid and 10- to 20-mm diameter PPLs, whereas the CT-TTNB group demonstrated more ground-glass and < 10-mm diameter PPLs. Both groups showed high tool-in-lesion confirmation rates (96.5% CBCT-GB vs 98.2% CT-TTNB; *P* = .130), whereas the CBCT-GB group showed more PPLs with a bronchus sign (40.1% vs 12.3%; *P* < .001).

**Table 2:** Patient and Nodule Characteristics

Characteristic	CBCT-GB (n = 392)	CT-TTNB (n = 503)	*P* Value
Patients^a^			
Sex			.892
Male	194 (50.5)	256 (51.1)	
Female	190 (49.5)	245 (48.9)	
Race and ethnicity			.010^b^
White, non-Hispanic	212 (55.2)	241 (48.1)	
Asian	114 (29.7)	145 (28.9)	
Other^c^	58 (15.1)	115 (23.0)	
Smoking history			.005
Current or former	213 (55.5)	230 (45.9)	
Never	171 (44.5)	271 (54.1)	
Cancer history			< .001^d^
NSCLC	64 (16.2)	76 (14.9)	
SCLC	1 (0.3)	2 (0.4)	
Extrathoracic	161 (40.8)	333 (65.2)	
None	169 (42.8)	100 (19.6)	
Age, y	70 (62.5-77.5)	69 (61-77)	.233
BMI, kg/m^2^	25.1 (21.8-28.4)	24.8 (21.5-28.2)	.682
Nodules			
Density			< .001^e^
Solid	302 (77.0)	410 (81.5)	
Part solid	78 (19.9)	60 (11.9)	
Ground glass	12 (3.1)	33 (6.6)	
Lobar location			.080
Right upper	114 (29.1)	121 (24.1)	
Right middle	38 (9.7)	33 (6.6)	
Right lower	72 (18.4)	114 (22.7)	
Left upper	69 (17.6)	79 (15.7)	
Lingula	24 (6.1)	38 (7.6)	
Left lower	75 (19.1)	118 (23.5)	
Longest diameter, mm			< .001^f^
< 10	22 (5.6)	58 (11.5)	
10-20	193 (49.2)	192 (38.2)	
20-30	89 (22.7)	124 (24.7)	
≥ 30	88 (22.4)	129 (25.6)	
Peripheral distribution			.264
Inner third of lung	51 (13.0)	53 (10.5)	
Middle third of lung	114 (29.1)	133 (26.4)	
Outer third of lung	227 (57.9)	317 (63.0)	
Bronchus sign present^g^	157 (40.1)	62 (12.3)	< .001
TIL confirmed^h^	362 (96.5)	494 (98.2)	.130

Data are presented as No. (%) or median (interquartile range) unless otherwise indicated. Categorial variables were compared using the Fisher exact test with a Bonferroni correction for subanalyses. Continuous variables were compared using either the Mann-Whitney *U* test or median test, as appropriate. CBCT-GB = cone beam CT imaging-guided bronchoscopy; CT-TTNB = CT imaging-guided transthoracic needle biopsy; NSCLC = non-small cell lung carcinoma; SCLC = small cell lung carcinoma; TIL = tool in lesion.

a Patient demographic percents are based on n = 384 for the CBCT-GB group and n = 501 for the CT-TTNB so as not to duplicate information for patients who had > 1 PPL biopsied during the same procedure.

b The statistical difference was driven by differences in ethnicities in the White non-Hispanic and other categories.

c Race/ethnicity other than white, non-Hispanic and Asian.

d The statistical difference was driven by differences in the extrathoracic and none categories.

e The statistical difference was driven by differences in the part solid and ground glass categories.

f The statistical difference was driven by differences in the < 10 and 10-20 categories.

g The bronchus sign is present when a bronchial airway is found to terminate in or extend through a lung nodule on CT imaging.

h When the biopsy tool is seen to be within the lesion on an intraoperative CT image.

### Diagnostic Outcomes

Diagnostic accuracy at 24 months was 90.7% (340/375) for the CBCT-GB group and 92.6% (440/475) for the CT-TTNB group (*P* = .301; OR, 0.979; 95% CI, 0.939-1.020). Among patients with a nonspecific result on index pathologic findings, 34.2% (26/76) in the CBCT-GB group and 23.6% (21/89) in the CT-TTNB group were reclassified as having malignant results at 24 months (*P* = .166) (Table 1, Table 3, 3, e-Table 1). Likelihood ratios for predicting future malignancy based on each nonspecific index pathologic result are detailed in Table 4.

**Table 3:** Primary and Secondary Outcomes

Outcome	CBCT-GB (n = 392)	CT-TTNB (n = 503)	*P* Value	OR^a^ (95% CI)
Primary outcomes				
Diagnostic accuracy at 24-mo follow-up^b^	340/375 (90.7)	440/475 (92.6)	.301	0.979 (0.939-1.020)
Probability of malignancy at 24-mo follow-up given nonspecific results on index pathologic analysis	26/76 (34.2)	21/89 (23.6)	.166	1.450 (0.891-2.359)
Lost to follow-up	17/392 (4.3)	28/503 (5.6)	.444	0.779 (0.433-1.403)
Secondary outcomes				
DA at 12-mo follow-up^b^	347/384 (90.4)	443/479 (92.5)	.266	0.977 (0.937-1.018)
DY per ATS and CHEST guidelines^b^	325/392 (82.9)	428/503 (85.1)	.376	0.974 (0.920-1.032)
Secondary procedures				
Subsequent invasive diagnostic procedure^c^	27/392 (6.9)	32/503 (6.4)	.787	1.083 (0.660-1.776)
Patients who met criteria for invasive mediastinal lymph node staging (cEBUS) at time of index biopsy^d^	111/392 (28.3)	93/503 (18.5)	< .001	1.532 (1.203-1.950)
Of these patients, no. who received cEBUS	96/111 (86.5)	13/93 (14.0)	< .001	6.187 (3.717-10.298)
Of those who received cEBUS, No. whose lymph nodes showed positive results for malignancy	7/96 (7.3)	5/13 (38.5)	.005	0.190 (0.070-0.511)

Radiation exposure^e^	Nonrobotic (n = 116)	Robotic (n = 276)			Median Difference (95% CI)
Median effective dose, mSv^f^	9.4 (4.4-14.4)		7.5 (4.7-10.2)	< .001^f^	1.8 (1.1-2.6)
	13.6 (7.4-19.9)	8.6 (4.4-12.8)			NA
Fluoroscopy time, min	19.0 (12.0-26.0)	13.0 (9.0-17.0)	NA	< .001	6.0 (4.0-8.0)
CT spins, No.	4.0 (2.6-5.4)	3.5 (2.6-4.9)	NA	< .001	1.0 (0.0-1.0)
Absorbed dose, mGy	263.0 (143.5-382.5)	174.5 (83.5-265.5)	NA	.002	68.0 (35.0-101.0)
DAP, Gy × cm^2^	85.3 (46.3-124.3)	53.7 (27.3-80.1)	NA	< .001	24.0 (12.0-36.0)
DAP per CT spin, Gy × cm^2^	18.3 (8.4-28.1)	17.0 (12.1-21.9)	NA	.071	3.1 (0.5-5.8)
Effective dose per CT spin, mSv	2.9 (1.4-4.5)	2.7 (1.9-3.5)	NA	.071	0.5 (0.1-0.9)

Data are presented as No./Total No. (%) or median (interquartile range) unless otherwise indicated. ATS = American Thoracic Society; CBCT-GB = cone beam CT imaging-guided biopsy; CHEST = American College of Chest Physicians; cEBUS = convex endobronchial ultrasound; CT-TTNB = CT imaging-guided transthoracic needle biopsy; DA = diagnostic accuracy; DAP = dose area product; DY = diagnostic yield; NA = not applicable; PPL = peripheral pulmonary lesion.

a Shown are the OR of CBCT-GB group relative to the CT-TTNB group giving the outcome of interest.

b See Table 1 for varying definitions of DA and DY.

c A subsequent invasive diagnostic procedure is any procedure that involved biopsy of the same PPL, another PPL, or cEBUS of mediastinal lymph nodes.

d Patients suspected of having malignancy without extrathoracic metastases who also have either a PPL in central two-thirds of lung fields, PPL size of ≥ 3 cm, or have enlarged or fluorodeoxyglucose-avid mediastinal or hilar lymph nodes.^35^^,^^36^

e Radiation exposure was recorded as DAP in the CBCT-GB group and dose length product (DLP) in the CT-TTNB group. To facilitate comparison, DAP was converted to effective dose using a factor of 0.16 mSv/Gy **×** cm^2^ whereas DLP was converted using 0.014 mSv/mGy, consistent with society recommendations.^27^, ^28^, ^29^, ^30^, ^31^

f Statistically significant at *P* < .001 when comparing all groups (nonrobotic CBCT-GB with robotic CBCT-GB with CT-TTNB). However, subanalyses showed that nonrobotic CBCT-GB vs robotic CBCT-GB (*P* = .001) and nonrobotic CBCT-GB vs CT-TTNB (*P* = .000) retained statistical significance, whereas robotic CBCT-GB vs CT-TTNB (*P* = .074) showed no difference.

**Table 4:** Likelihood Ratio for a Future Malignant Result at the 24-Month Follow-Up Given a Nonspecific Index Pathologic Result

Index Pathologic Result	CBCT-GB (n = 95)		CT-TTNB (N = 121)		Combined (CBCT-GB or CT-TTNB; n = 216)	
	No.	Likelihood Ratio (95% CI)	No.	Likelihood Ratio (95% CI)	No.	Likelihood Ratio (95% CI)
Organizing pneumonia	19	0.057 (0.013-0.249)	24	0.041 (0.010-0.181)	43	0.048 (0.017-0.136)
Fibroelastotic scar	19	0.062 (0.014-0.270)	12	0.116 (0.030-0.439)	31	0.081 (0.031-0.214)
Granulomatous inflammation	9	0.000^a^	22	0.017 (0.002-0.126)	31	0.015 (0.002-0.109)
Atypical cells	11	Infinity^b^	17	0.539 (0.189-1.541)	28	1.300 (0.494-3.420)
Necrosis	3	0.805 (0.074-8.780)	8	0.108 (0.021-0.547)	11	0.217 (0.062-0.760)
Nonspecific inflammation	13	0.045 (0.006-0.348)	26	0.016 (0.002-0.118)	39	0.025 (0.006-0.104)
Normal lung tissue	21	0.517 (0.198-1.353)	12	0.108 (0.021-0.547)	33	0.298 (0.133-0.665)

CBCT-GB = cone beam CT imaging-guided bronchoscopy; CT-TTNB = CT imaging-guided transthoracic needle biopsy.

a Implies that none of the index pathologic results of granulomatous inflammation in the CBCT-GB group were found to be malignant on 24-mo follow-up.

b Implies that all of the index pathologic results of atypical cells in the CBCT-GB group were found to be malignant on 24-mo follow-up.

Secondary outcomes are detailed in Table 3 and e-Appendix 1. The American Thoracic Society and CHEST DY was 82.9% (325/392) for CBCT-GB and 85.1% (428/503) for CT-TTNB (*P* = .376; OR, 0.974; 95% CI, 0.920-1.032). Across all DA and DY definitions, no significant between-group differences were observed. Among patients meeting criteria for upfront invasive mediastinal lymph node staging (suspected malignant PPL without extrathoracic metastases plus either size of ≥ 3 cm, central location, or enlarged or PET-avid hilar or mediastinal nodes),^37^^,^^38^ 86.5% of patients in the CBCT-GB group underwent staging concurrently with PPL biopsy, whereas 14% of patients in the CT-TTNB group required a separate subsequent procedure (*P* < .001). Malignant nodal involvement was confirmed in 7.3% vs 38.5% of patients, respectively (*P* = .005). The median effective radiation dose was 1.8 mSv higher for the CBCT-GB group (*P* < .001). However, a subanalysis showed no difference when specifically comparing RAB CBCT-GB with CT-TTNB (median difference, 1.1 mSv; *P* = .074), suggesting that the overall difference in radiation exposure was driven by higher radiation use in the non-RAB CBCT-GB procedures.

Procedural times differed significantly (Table 5), with a median of 67 minutes for CBCT-GB vs 31 minutes for CT-TTNB (median difference, 35 minutes; *P* < .001). Subanalyses comparing patients who underwent CT-TTNB with those who underwent CBCT-GB performed with or without RAB and with or without nodal staging consistently showed shorter times for CT-TTNB, with the smallest difference in time between RAB CBCT-GB without nodal staging (47 minutes) and CT-TTNB (31 minutes; median difference, 16 minutes; *P* < .001).

**Table 5:** Procedural Times

Group			*P* Value	Median Difference (95% CI)
CBCT-GB (n = 392)		CT-TTNB (n = 503)		
Total	67.0 (47.5-86.5)	31.0 (24.5-37.5)	< .001	35.0 (32.0-38.0)
Robotic			< .001^a^	NA
Without (n = 116)	88.5 (66.5-110.5)			
With (n = 276)	59.5 (44.0-75.0)			
cEBUS			< .001^a^	NA
Without (n = 135)	50.0 (34.5-65.5)			
With (n = 257)	76.0 (58.5-93.5)			
Combination			< .001^b^	NA
Without robot and without cEBUS (n = 32)	67.5 (17.0-84.5)			
Without robot and with cEBUS (n = 84)	96.0 (19.5-115.5)			
With robot and without cEBUS (n = 103)	47.0 (34.0-60.0)			
With robot and with cEBUS (n = 173)	69.0 (54.0-84.0)			

CBCT-GB = cone beam CT imaging-guided biopsy; cEBUS = convex endobronchial ultrasound; CT-TTNB = CT imaging-guided transthoracic needle biopsy; NA = not applicable.

a All pairwise group combinations also are statistically significant at *P* = .050.

b All pairwise group combinations are statistically significant at *P* = .050, except when CBCT-GB with robot and with cEBUS is compared with CBCT-GB without robot and without cEBUS (*P* = .723).

Procedural complications occurred in 4.3% of patients undergoing CBCT-GB and 41.6% of patients undergoing CT-TTNB (*P* < .001). Pneumothorax rates were 1.8% for CBCT-GB and 31.4% for CT-TTNB (*P* < .001), with 0.5% and 5.6% of patients requiring intervention, respectively. Severe bleeding or cardiorespiratory failure occurred in 3.3% of patients undergoing CBCT-GB and 6.0% of patients undergoing CT-TTNB (*P* < .001). One death occurred in the CBCT-GB group and no deaths occurred in the CT-TTNB group (Table 6).

**Table 6:** Safety Outcomes

Complication^a^	Cone Beam CT Imaging-Guided Biopsy (n = 392)	CT Imaging-Guided Transthoracic Needle Biopsy (n = 503)	*P* Value
Any complication	17 (4.3)	209 (41.6)	< .001
Pneumothorax			
Without intervention^b^	5 (1.3)	130 (25.8)	< .001
With intervention^b^	2 (0.5)	28 (5.6)	< .001
Serious^c^	13 (3.3)	30 (6.0)	< .001
Death	1 (0.3)	0 (0.0)	1.000

Data are presented as No. (%) unless otherwise indicated.

a Individual complication numbers may surpass total No. of complications because a patient could have > 1 type of complication.

b Defined as anything other than observation of the pneumothorax. Such interventions included any of the following: needle aspiration, pigtail catheter placement, or surgical chest tube placement.

c Defined as any degree of bleeding requiring blood transfusion or hospital admission, respiratory failure necessitating the use of noninvasive or invasive ventilation, impending cardiac compromise requiring vasopressor support, or overnight hospital admission for any reason.

Malignancy prevalence was 75.5% overall (71.3% in CBCT-GB; 78.8% in CT-TTNB). Sensitivity analyses showed no between-group differences in DA or sensitivity for malignancy (e-Table 2). In univariate analyses of the primary outcome stratified by patient and PPL characteristics, lobar location and bronchus sign seemed potentially to modify the effect of biopsy method on DA (Table 7).

**Table 7:** Univariate Analysis of Primary Outcome (Diagnostic Accuracy at 24-Month Follow-up) by Subgroup Stratification

Data are presented at No./Total No. (%) unless otherwise indicated. CBCT-GB = cone beam CT imaging-guided bronchoscopy; CT-TTNB = CT imaging-guided transthoracic needle biopsy; PPL = peripheral pulmonary lesion.

^a^OR of CBCT-GB relative to CT-TTNB giving a diagnostic result stratified by patient and PPL characteristics; crude OR, 0.979 (95% CI, 0.939-1.020), vertical dashed line. The ORs were obtained before performing binary logistic regression modeling to check for the presence of potential effect modifiers.

^b^The bronchus sign is present when a bronchial airway is found to terminate in or extend through a lung nodule on CT imaging.

**Figure 2:**
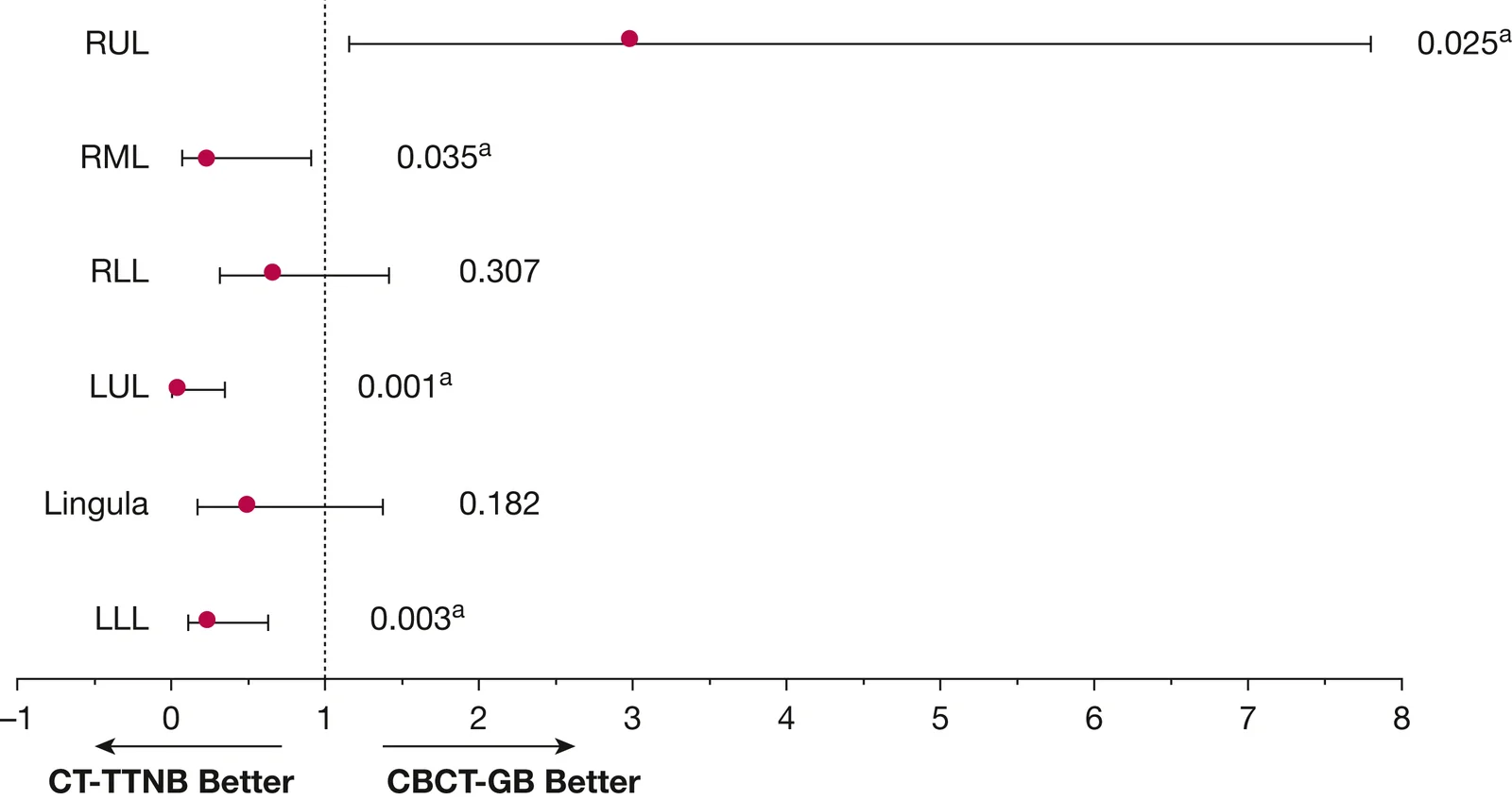
Graph showing lobe-specific ORs for diagnostic success after adjusting for peripheral pulmonary lesion (PPL) density, size, peripheral location, and bronchus sign. Each interval is centered around the adjusted OR of CBCT-GB group relative to CT-TTNB group giving a diagnostic result per PPL lobar location with 95% CI. ^a^Statistically significant results (P values). Adjusted OR and *P* values calculated using the delta method from estimated marginal means. CBCT-GB = cone beam CT imaging-guided bronchoscopy; CT-TTNB = CT imaging-guided transthoracic needle biopsy; LLL = left lower lobe; LUL = left upper lobe; RLL = right lower lobe; RML = right middle lobe; RUL = right upper lobe.

In a multivariable interaction model (e-Table 3) adjusting for PPL density, size, peripheral location, and bronchus sign, lobar location significantly modified the relationship between biopsy method and DA (*P* = .019), whereas bronchus sign did not (*P* = .072). CBCT-GB was associated with higher odds of success in the right upper lobe (adjusted OR, 3.000; *P* = .025). CT-TTNB significantly outperformed CBCT-GB in the right middle lobe (OR, 0.257; *P* = .035), left upper lobe (OR, 0.060; *P* = .001), and left lower lobe (OR, 0.259; *P* = .003). No significant differences were observed in the right lower lobe or lingula (*P* = .307 and *P* = .182, respectively). Lobe-specific adjusted ORs and predicted probabilities are presented in Table 8 and Figure 2. The study was underpowered to evaluate effect measure modification on sensitivity for malignancy or to assess interactions stratified by nonrobotic vs robotic CBCT-GB compared with CT-TTNB.

**Table 8:** Probability of Lobe-Specific Diagnostic Success After Adjusting for PPL Density, Size, Peripheral Location, and Bronchus Sign

PPL Lobar Location	Probability of Diagnostic Success, % (95% CI)^a^		Adjusted OR (95% CI)	*P* Value
	CBCT-GB	CT-TTNB		
RUL	96 (88-99)	88 (78-94)	3.000 (1.152-7.812)	.025
RML	82 (59-94)	95 (79-99)	0.257 (0.073-0.909)	.035
RLL	86 (71-94)	90 (80-96)	0.677 (0.323-1.420)	.307
LUL	83 (67-92)	99 (91-100)	0.060 (0.010-0.344)	.001
Lingula	71 (43-89)	83 (62-93)	0.499 (0.181-1.375)	.182
LLL	80 (65-90)	94 (85-98)	0.259 (0.107-0.626)	.003

CBCT-GB = cone beam CT imaging-guided bronchoscopy; CT-TTNB = CT imaging-guided transthoracic needle biopsy; LLL = left lower lobe; LUL = left upper lobe; PPL = peripheral pulmonary lesion; RLL = right lower lobe; RML = right middle lobe; RUL = right upper lobe.

a Probability of diagnostic success at the 24-mo follow-up stratified by PPL lobar location after adjusting for PPL density, size, peripheral location, and bronchus sign. Shown are the ORs of the CBCT-GB group relative to the CT-TTNB group of giving a diagnostic result per PPL lobar location. Adjusted ORs and *P* values were calculated using the delta method from estimated marginal means.

## Discussion

This study is the first to compare diagnostic and procedural outcomes between CBCT-GB and CT-TTNB for patients with PPLs. Early accurate diagnosis of PPLs is crucial for identifying patients who need cancer treatment vs those with benign conditions. The expediency of this process—from nodule identification to confirmatory diagnosis—is of particular importance and is recommended to be completed within 21 days because delays have been associated with pathologic upstaging and worse outcomes.^39^, ^40^, ^41^, ^42^, ^43^, ^44^, ^45^ Large studies comparing bronchoscopic with transthoracic biopsy methods are rare, except for the recent Navigation Endoscopy to Reach Indeterminate Lung Nodules Versus Transthoracic Needle Aspiration, A Randomized Controlled Study (VERITAS) trial, although it did not include the routine use of advanced multiaxial imaging such as CBCT imaging in the bronchoscopic arm.^23^ Our comparative study—the largest to date with 895 patients—suggests that CBCT-GB, which has a similar diagnostic accuracy as CT-TTNB, but offers improved safety and concurrent lymph node staging, may be the preferred initial procedure for PPL diagnosis.

The diagnostic accuracy (regardless of the definition used) was comparable in both groups and was consistent with previous estimates for these technologies.^4^^,^^13^, ^14^, ^15^, ^16^, ^17^, ^18^ When comparing the CBCT-GB DY with the DY of the navigational bronchoscopy arm in the VERITAS trial, our study showed slightly higher DY estimates (82.9% vs 79.3%).^23^ We believe this is attributable to the superior 3-dimensional imaging capabilities of intraoperative CBCT imaging coupled with augmented fluoroscopy. This aligns with prior data showing that the DA of procedures performed with radial EBUS or EMN-based navigation platforms is lower in comparison with those performed using CBCT imaging.^13^, ^14^, ^15^, ^16^, ^17^ The DA of the CT-TTNB group was consistent with that of previous studies.^4^

CBCT-GB was found to have 2 major advantages over CT-TTNB: improved safety and the ability to perform simultaneous lymph node staging. CBCT-GB showed a significantly lower incidence of procedural complications of any kind, supporting image-guided bronchoscopy as the safer method for PPL diagnosis.^5^^,^^6^^,^^23^ Additionally, more patients received appropriate and timely guideline-based care for invasive nodal staging when undergoing PPL biopsy via CBCT-GB as compared with those undergoing CT-TTNB.^37^^,^^38^ This discrepancy may be the result of ease of performing concomitant staging during CBCT-GB, established referral patterns to interventional radiology for CT-TTNB, and lack of knowledge among referring providers as to which patients necessitate upfront invasive nodal staging at the time of PPL biopsy. Although formal cost comparisons were not performed, prior data suggest that CBCT-GB also may be more cost-effective than CT-TTNB for patients who require invasive nodal staging.^46^

Our study showed that CBCT-GB performed with a RAB platform has similar radiation exposure as CT-TTNB, with both being comparable to the average effective dose for an abdominal CT scan.^29^ The improved radiation doses and shorter procedure times of RAB procedures likely are the result of the RAB platform’s superior maneuverability, reach, and stability. Methods to maintain the precise position of a conventional flexible bronchoscope are cumbersome, time-consuming, and error prone, thus leading to increased procedure times and radiation use.^13^ In contrast, robotic-assisted bronchoscopes readily maintain their position, obviating the need for adjunctive measures to ensure stability, thereby reducing the time and radiation dose needed to reach the target PPL.

Although CBCT-GB procedures typically took longer than CT-TTNB procedures, this was influenced heavily by the inclusion of patients using a nonrobotic bronchoscope and patients requiring nodal staging in the CBCT-GB group. When comparing the time needed for a PPL biopsy alone, RAB CBCT-GB procedures were only a median of 16 minutes longer than CT-TTNB procedures. Adding nodal staging to RAB CBCT-GB procedures increased the time by a median of 22 minutes, an increase undoubtedly shorter than the time it would take to schedule and perform a separate nodal staging procedure after CT-TTNB. By expediting this process from nodule identification to diagnosis and staging completion, RAB CBCT-GB likely reduces potential delays in care that could lead to inadvertent pathologic upstaging of the PPL.

About one-third of patients with nonspecific index pathologic results later were found to have malignancy. Likelihood ratio analysis showed atypical cells, necrosis, and normal lung tissue pathologic features largely were noninformative, whereas organizing pneumonia, granulomatous inflammation, fibroelastotic scar, and nonspecific inflammation likely substantially decreased malignancy probability after testing. Whether this is sufficient to classify organizing pneumonia and granulomatous inflammation as specifically benign according to American Thoracic Society and CHEST guidelines will require adequately powered future analyses, which also may clarify whether fibroelastotic scar and nonspecific inflammation merit inclusion. Although a larger sample size is needed to strengthen these findings, they support the notion that DA is not equivalent to DY.

An exploratory analysis showed that PPL lobar location was the only modifier of the relationship between biopsy method and diagnostic accuracy. CBCT-GB outperformed CT-TTNB in the right upper lobe, but underperformed in most left-sided lobes. Potential explanations include greater target localization challenges from cardiac motion on the left and dilution of CBCT-GB performance by inclusion of non-RAB procedures, because some data suggest that non-RAB procedures have lower diagnostic success than RAB procedures.^12^^,^^47^ This study was underpowered to disentangle these factors and should be followed by adequately powered, bronchoscopy platform-stratified future investigations before adopting lobe-specific procedural preferences.

### Strengths and Limitations

This study has several strengths. First, the sample size of 895 patients is significantly larger than previous studies, enabling detailed analyses on metrics such as procedural duration, radiation exposure, and outcomes of secondary procedures.^5^, ^6^, ^7^, ^8^, ^9^, ^10^, ^11^, ^12^^,^^21^^,^^23^ Second, the granular and transparent reporting of diagnostic accuracy definitions and outcome classifications facilitates easier and more meaningful comparisons with past and future studies.^19^^,^^20^^,^^24^ Third, multiple sensitivity analyses of the primary outcome corroborated the main analysis results, showing comparable diagnostic accuracies between CBCT-GB and CT-TTNB. Fourth, the long 24-month follow-up period with a low attrition rate provided crucial data to understand better the nature of nonspecific index pathologic results, because most missed malignant nodules would have grown significantly or been biopsied again during this time frame. Finally, the retrospective design reflects real-world practice and referral patterns because the study protocol did not dictate which patients were referred for which procedure, nor did it intentionally attempt to create balance between the 2 groups. However, institutional pathways likely influenced baseline differences, including higher smoking prevalence in the CBCT-GB group and greater preexisting extrathoracic malignancy in the CT-TTNB group.

This study also has several limitations. First, all patients were recruited from a single academic center and all procedures were performed by trained subspecialists using a standardized anesthetic and ventilatory protocol, which may limit generalizability across different practice settings and providers. Second, the use of rapid onsite cytologic evaluation was variable, reflecting real-world practice, but was documented poorly, limiting conclusions on its effect on DA. Third, all biopsies in the CBCT-GB group were performed using 2 different CBCT imaging systems and the same RAB platform after its implementation, limiting generalizability to other CBCT imaging and RAB systems. Other CBCT imaging systems may have different image qualities, spin times, and augmented fluoroscopic capabilities (especially mobile systems), whereas other RAB platforms may have different degrees of maneuverability, stability, and navigational reliability. Fourth, although radiation dose conversions to effective dose were performed using recommend conversion factors,^27^, ^28^, ^29^, ^30^, ^31^ these are debated, and alternative factors could yield different estimates. Fifth, cost-effectiveness was not assessed. Finally, despite the large sample size, each nonspecific index pathologic result contained a small number of patients, so likelihood ratios for future malignancy risk should be interpreted cautiously and investigated further in larger studies.

## Interpretation

This large and, to our knowledge, first-of-its-kind study suggests that CBCT-GB has a comparable 24-month diagnostic accuracy as CT-TTNB while offering improved safety and concurrent nodal staging, supporting CBCT-GB as the preferred initial approach for PPL diagnosis in appropriately resourced centers. Future research should evaluate this conclusion in pragmatic randomized trials across diverse CBCT imaging and RAB platforms to assess cost-effectiveness, the usefulness of rapid onsite cytologic evaluation, and the optimal management of nonspecific index pathologic results.

## Funding/Support

The authors have reported to *CHEST* that no funding was received for this study.

## Financial/Nonfinancial Disclosures

The authors have reported to *CHEST* the following: D. E. O. is a paid consultant for Intuitive. H. B. received an academic grant from Philips. D. F. performs education work with Johnson and Johnson. P. P. and B. S. are paid consultants for Johnson and Johnson. J. C. is a paid consultant for Johnson and Johnson and an educational consultant for PulmonX. None declared (A. Y., H. J. C., C.-Y. K., E. S., C. C.).

## Figures and Tables

**Figure S1:**
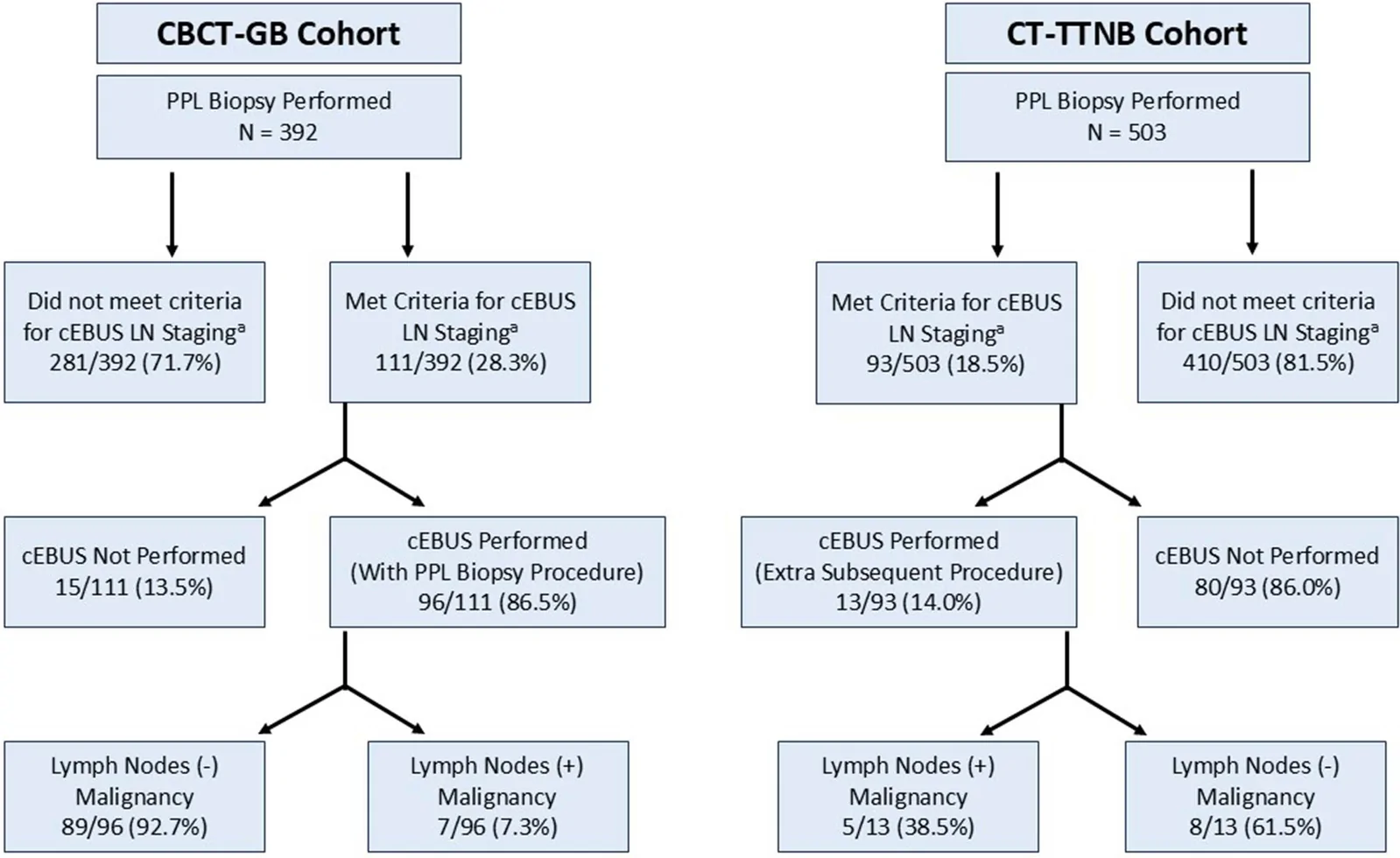
Flow Diagram showing convex EndoBronchial UltraSound use for lymph node biopsies and outcomes. ^a^Patients suspected of having malignancy without extra-thoracic metastases who also have either a PPL in central two thirds of lung fields, PPL size >= 3cm, or have enlarged or FDG avid mediastinal or hilar lymph nodes^37, 38^. CBCT-GB: Cone Beam Computed Tomography-Guided Bronchoscopy; CT-TTNB: Computed Tomography-Guided TransThoracic Needle Biopsy; cEBUS: Convex EndoBronchial UltraSound; PPL: Peripheral Pulmonary Lesion; LN: Lymph Node.

## Supplementary Data

e-Online Data
