# Supplementary material for: Cone Beam CT Imaging-Guided Bronchoscopy vs CT Imaging-Guided Transthoracic Needle Biopsy for Peripheral Pulmonary Lesion Diagnosis
Source: Chest. 2026 Mar 25;170(2):604–18. doi: 10.1016/j.chest.2026.02.038 (PMC13494254; doi:10.1016/j.chest.2026.02.038)
Supplement: Supplementary file 1 [file mmc1.docx]

**Title:**	Cone Beam Computed Tomography-Guided Bronchoscopy versus Computed Tomography-Guided Transthoracic Needle Biopsy for Peripheral Pulmonary Lesion Diagnosis

**SUPPLEMENTARY APPENDIX**

**Tables Referenced in Manuscript**

*e-Table 1	4-5*

High Order Contingency Tables for Primary Outcome of Diagnostic Accuracy at 24 months of follow up

*e-Table 2	6*

Sensitivity Analyses of the Primary Outcome (Diagnostic Accuracy at 24 month follow up)

*e-Table 3	7*

Final Binary Logistic Regression Model with Interaction Terms for 24 month Diagnostic Accuracy

*e-Table 4	8*

Probability of Lobe-Specific Diagnostic Success after adjusting for PPL density, size, peripheral location, and bronchus sign (Calculated using parameter estimates and the model-based covariance matrix)

**Non-Robotic CBCT-GB, Robotic CBCT-GB, CT-TTNB Subgroup Comparisons**

*e-Table 5	10*

Patient and Nodule Characteristics *(Non-Robotic CBCT-GB, Robotic CBCT-GB, CT-TTNB)*

*e-Table 6	11*

Primary and Secondary Outcomes (Non-Robotic CBCT-GB, Robotic CBCT-GB, CT-TTNB)

*e-Table 7	11*

Safety Outcomes *(Non-Robotic CBCT-GB, Robotic CBCT-GB, CT-TTNB)*

*e-Table 8	12*

Univariate Analysis of Primary Outcome *of* Diagnostic Accuracy at 24 month follow up *(Non-Robotic CBCT-GB, Robotic CBCT-GB, CT-TTNB)*

**High Order Contingency Tables**

*e-Table 9	14-15*

High Order Contingency Tables for Primary Outcome of Diagnostic Accuracy at 24 months of follow up *(Non-Robotic CBCT-GB versus Robotic CBCT-GB)*

*e-Table 10	16*

High Order Contingency Tables for classification of infection type after 24 months of follow up (CBCT-GB versus CT-TTNB)

*e-Table 11	17*

High Order Contingency Tables for classification of infection type after 24 months of follow up *(Non-Robotic CBCT-GB versus Robotic CBCT-GB)*

*e-Table 12	18-19*

High Order Contingency Tables for Secondary Outcome of Diagnostic Accuracy at 12 months of follow up

*e-Table 13	20-21*

High Order Contingency Tables for Secondary Outcome of Diagnostic Accuracy at 12 months of follow up *(Non-Robotic CBCT-GB versus Robotic CBCT-GB)^a^*

*e-Table 14	22*

High Order Contingency Tables for classification of infection type after 12 months of follow up (CBCT-GB versus CT-TTNB)

*e-Table 15	23*

High Order Contingency Tables for classification of infection type after 12 months of follow up *(Non-Robotic CBCT-GB versus Robotic CBCT-GB)^a^*

**Other Tables and Figures**

*e-Table 16	25*

Diagnostic Accuracy and Diagnostic Yield Stratified by Tool-In-Lesion Confirmation

*e-Figure 1	26*

Flow Diagram showing convex EndoBronchial UltraSound use for lymph node biopsies and outcomes

*e-Figure 2	27*

Statistical Analysis Flow Diagram

**TABLES REFERENCED IN MANUSCRIPT**

**e-Table 1. High Order Contingency Tables for Primary Outcome of Diagnostic Accuracy at 24 months of follow up^a^**

| **Cone Beam Computed Tomography Guided Biopsy** | | **Reference Standard of Truth Result** | | | | | | | | | | | |
| --- | --- | --- | --- | --- | --- | --- | --- | --- | --- | --- | --- | --- | --- |
|  |  | NSCLC | SCLC | Pulmonary Carcinoid | Metastases to Lung | Infection | Benign Nodule | Sarcoid | Stable >=24 months | Shrank or Resolved | Enlarged | <24 months follow up | Total |
| **Index Pathology Result** | NSCLC | 177 | 0 | 0 | 0 | 0 | 0 | 0 | 0 | 0 | 0 |  | 177 |
|  | SCLC | 0 | 0 | 0 | 0 | 0 | 0 | 0 | 0 | 0 | 0 | 0 | 0 |
|  | Pulmonary Carcinoid | 0 | 0 | 9 | 0 | 0 | 0 | 0 | 0 | 0 | 0 | 0 | 9 |
|  | Metastases to Lung  ***Diagnostic*** | 0 | 0 | 0 | 54 | 0 | 0 | 0 | 0 | 0 | 0 | 0 | 54 |
|  | Infection | 0 | 0 | 0 | 0 | 48 | 0 | 0 | 0 | 0 | 0 | 0 | 48 |
|  | Benign Nodule | 0 | 0 | 0 | 0 | 0 | 9 | 0 | 0 | 0 | 0 | 0 | 9 |
|  | Organizing Pneumonia^b^  ***Varies^b^*** | 1 | 0 | 0 | 1 | 0 | 0 | 0 | 2 | 12 | 0 | 3 | 19 |
|  | Granulomatous Inflammation^b^ | 0 | 0 | 0 | 0 | 0 | 0 | 2 | 2 | 2 | 0 | 3 | 9 |
|  | Fibroelastotic Scar | 2 | 0 | 0 | 0 | 0 | 0 | 0 | 9 | 4 | 0 | 4 | 19 |
|  | Necrosis | 0 | 0 | 0 | 2 | 1 | 0 | 0 | 0 | 0 | 0 | 0 | 3 |
|  | Non-Specific Inflammation | 0 | 0 | 0 | 1 | 2 | 1 | 0 | 2 | 4 | 1 | 2 | 13 |
|  | Atypical Cells  ***Non-Diagnostic*** | 8 | 0 | 0 | 2 | 0 | 0 | 0 | 0 | 0 | 0 | 1 | 11 |
|  | Non-Specific Normal Lung Tissue | 4 | 0 | 1 | 4 | 0 | 0 | 0 | 6 | 1 | 1 | 4 | 21 |
|  | Total | 192 | 0 | 10 | 64 | 51 | 10 | 2 | 21 | 23 | 2 | 17 | 392 |
| **Computed Tomography Guided TransThoracic Needle Biopsy** | | **Reference Standard of Truth Result** | | | | | | | | | | | |
|  |  | NSCLC | SCLC | Pulmonary Carcinoid | Metastases to Lung | Infection | Benign Nodule | Sarcoid | Stable >=24 months | Shrank or Resolved | Enlarged | <24 months follow up | Total |
|  | NSCLC | 131 | 0 | 0 | 0 | 0 | 0 | 0 | 0 | 0 | 0 | 0 | 131 |
|  | SCLC | 0 | 7 | 0 | 0 | 0 | 0 | 0 | 0 | 0 | 0 | 0 | 7 |
|  | Pulmonary Carcinoid | 0 | 0 | 1 | 0 | 0 | 0 | 0 | 0 | 0 | 0 | 0 | 1 |
|  | Metastases to Lung | 0 | 0 | 0 | 211 | 0 | 0 | 0 | 0 | 0 | 0 | 0 | 211 |
|  | Infection | 0 | 0 | 0 | 0 | 26 | 0 | 0 | 0 | 0 | 0 | 0 | 26 |
|  | Benign Nodule  **Index Pathology Result**  ***Diagnostic***  ***Non-Diagnostic***  ***Varies^b^*** | 0 | 0 | 0 | 0 | 0 | 6 | 0 | 0 | 0 | 0 | 0 | 6 |
|  | Organizing Pneumonia^b^ | 0 | 0 | 0 | 2 | 4 | 0 | 0 | 1 | 8 | 2 | 7 | 24 |
|  | Granulomatous Inflammation^b^ | 1 | 0 | 0 | 0 | 0 | 0 | 2 | 7 | 7 | 0 | 5 | 22 |
|  | Fibroelastotic Scar | 1 | 0 | 0 | 2 | 0 | 0 | 0 | 6 | 1 | 0 | 2 | 12 |
|  | Necrosis | 0 | 0 | 0 | 2 | 2 | 1 | 0 | 0 | 2 | 1 | 0 | 8 |
|  | Non-Specific Inflammation | 0 | 0 | 0 | 1 | 3 | 0 | 0 | 5 | 9 | 0 | 8 | 26 |
|  | Atypical Cells | 9 | 0 | 0 | 1 | 1 | 0 | 0 | 3 | 1 | 0 | 2 | 17 |
|  | Non-Specific Normal Lung Tissue | 0 | 0 | 0 | 2 | 1 | 1 | 0 | 1 | 2 | 1 | 4 | 12 |
|  | Total | 142 | 7 | 1 | 221 | 37 | 8 | 2 | 23 | 30 | 4 | 28 | 503 |
| **Any Biopsy Method (CBCT-GB or CT-TTNB)** | | **Reference Standard of Truth Result** | | | | | | | | | | | |
|  |  | NSCLC | SCLC | Pulmonary Carcinoid | Metastases to Lung | Infection | Benign Nodule | Sarcoid | Stable >=24 months | Shrank or Resolved | Enlarged | <24 months follow up | Total |
|  | NSCLC | 308 | 0 | 0 | 0 | 0 | 0 | 0 | 0 | 0 | 0 | 0 | 308 |
|  | SCLC | 0 | 7 | 0 | 0 | 0 | 0 | 0 | 0 | 0 | 0 | 0 | 7 |
|  | Pulmonary Carcinoid | 0 | 0 | 10 | 0 | 0 | 0 | 0 | 0 | 0 | 0 | 0 | 10 |
|  | Metastases to Lung | 0 | 0 | 0 | 265 | 0 | 0 | 0 | 0 | 0 | 0 | 0 | 265 |
|  | Infection | 0 | 0 | 0 | 0 | 74 | 0 | 0 | 0 | 0 | 0 | 0 | 74 |
|  | Benign Nodule | 0 | 0 | 0 | 0 | 0 | 15 | 0 | 0 | 0 | 0 | 0 | 15 |
|  | Organizing Pneumonia^b^ | 1 | 0 | 0 | 3 | 4 | 0 | 0 | 3 | 20 | 2 | 10 | 43 |
|  | Fibroelastotic Scar^b^  ***Varies^b^***  ***Non-Diagnostic***  ***Diagnostic***  **Index Pathology Result** | 3 | 0 | 0 | 2 | 0 | 0 | 0 | 15 | 5 | 0 | 6 | 31 |
|  | Granulomatous Inflammation | 1 | 0 | 0 | 0 | 0 | 0 | 4 | 9 | 9 | 0 | 8 | 31 |
|  | Atypical Cells | 17 | 0 | 0 | 3 | 1 | 0 | 0 | 3 | 1 | 0 | 3 | 28 |
|  | Necrosis | 0 | 0 | 0 | 4 | 3 | 1 | 0 | 0 | 2 | 1 | 0 | 11 |
|  | Non-Specific Inflammation | 0 | 0 | 0 | 2 | 5 | 1 | 0 | 7 | 13 | 1 | 10 | 39 |
|  | Non-Specific Normal Lung Tissue | 4 | 0 | 1 | 6 | 1 | 1 | 0 | 7 | 3 | 2 | 8 | 33 |
|  | Total | 334 | 7 | 11 | 285 | 88 | 18 | 4 | 44 | 53 | 6 | 45 | 895 |

NSCLC: Non-small cell lung carcinoma; SCLC: Small cell lung carcinoma; CBCT-GB: Cone Beam Computed Tomography Guided Bronchoscopy; CT-TTNB: Computed Tomography Guided TransThoracic Needle Biopsy; ATS-ACCP: American Thoracic Society-American College of Chest Physicians; DY: Diagnostic Yield; DA: Diagnostic Accuracy.

^a^This table shows into what category every index pathology result was put into after 24 months of follow up data, and forms the basis from which diagnostic accuracy, diagnostic yield and other results in this paper are calculated. For further clarity on definitions of diagnostic accuracy, diagnostic yield and other terms, see Table 1.

^b^Organizing Pneumonia and Granulomatous Inflammation are considered diagnostic on index pathology per the ATS-ACCP definition of DY^26^. Both are considered non-diagnostic on index pathology per the definitions of DA employed in this paper.

**e-Table 2. Sensitivity Analyses of the Primary Outcome (Diagnostic Accuracy at 24 month follow up)**

| Sensitivity Cohort^a^ | Outcome at 24 month follow up | Cone Beam Computed Tomography Guided Biopsy (N = 392) | Computed Tomography Guided TransThoracic Needle Biopsy (N = 503) | *P*-Value | Odds Ratio^b^  (95% CI) |
| --- | --- | --- | --- | --- | --- |
|  |  | No. Patients/Total No. (%) | |  |  |
| Primary Outcome^c^  (LTFU excluded from analysis) | Diagnostic Accuracy (Overall) | 340/375 (90.7) | 440/475 (92.6) | 0.301 | 0.979 (0.939 - 1.020) |
|  | Diagnostic Accuracy for Malignancy | 347/373 (93.0) | 450/471 (95.5) | --- | --- |
|  | Prevalence for Malignancy | 266/373 (71.3) | 371/471 (78.8) | --- | --- |
|  | Sensitivity for Malignancy (%) | 240/266 (90.2) | 350/371 (94.3) | 0.653 | --- |
| Index Pathology Non-Specific Results that enlarged or were LTFU considered true negative for malignancy (Diagnostic) | Diagnostic Accuracy (Overall) | 359/392 (91.6) | 472/503 (93.8) | 0.194 | 0.976 (0.940 - 1.013) |
|  | Diagnostic Accuracy for Malignancy | 366/392 (93.4) | 482/503 (95.8) | --- | --- |
|  | Prevalence for Malignancy | 266/392 (67.9) | 371/503 (73.8) | --- | --- |
|  | Sensitivity for Malignancy (%) | 240/266 (90.2) | 350/371 (94.3) | 0.414 | --- |
| Index Pathology Non-Specific Results that enlarged or were LTFU considered false negative for malignancy (Non-Diagnostic) | Diagnostic Accuracy (Overall) | 340/392 (86.7) | 440/503 (87.5) | 0.743 | 0.992 (0.942 - 1.043) |
|  | Diagnostic Accuracy for Malignancy | 347/392 (88.5) | 450/503 (89.5) | --- | --- |
|  | Prevalence for Malignancy | 285/392 (72.7) | 403/503 (80.1) | --- | --- |
|  | Sensitivity for Malignancy (%) | 240/285 (84.2) | 350/403 (86.8) | 0.346 | --- |

LTFU: Lost to Follow Up referring to patients who did not have 24 months of follow up data; CBCT-GB: Cone Beam Computed Tomography Guided Bronchoscopy; CT-TTNB: Computed Tomography Guided TransThoracic Needle Biopsy, OR: Odds Ratio.

^a^Specificity for Malignancy was 100% in all groups.

^b^Shown are the OR of CBCT-GB Group relative to CT-TTNB Group giving the outcome of interest.

^c^For the primary outcome, diagnostic accuracy considered enlarging lesions as non-diagnostic while those LTFU were excluded. For all other metrics in this cohort, both enlarging lesions and those LTFU were excluded.

**e-Table 3. Final Binary Logistic Regression Model with Interaction Terms for 24 month Diagnostic Accuracy**

| Variable in the Equation^a^ | Coefficient (B) | *P*-Value | Exp (B) with 95% CI |
| --- | --- | --- | --- |
| Constant | 1.758 | 0.004 | 5.803 |
|  |  |  |  |
| Biopsy Method |  |  |  |
| CT-TTNB (reference) | 0 (ref) |  | 1 (ref) |
| CBCT-GB | 1.156 | 0.090 | 3.179 (0.836 – 12.092) |
|  |  |  |  |
| PPL Lobar Location |  | 0.133 |  |
| RUL (reference) | 0 (ref) |  | 1 (ref) |
| RML | 0.838 | 0.308 | 2.312 (0.461 – 11.606) |
| RLL | 0.201 | 0.676 | 1.223 (0.475 – 3.150) |
| LUL | 2.386 | 0.027 | 10.873 (1.313 – 90.055) |
| Lingula | -0.445 | 0.462 | 0.641 (0.196 – 2.097) |
| LLL | 0.707 | 0.186 | 2.028 (0.711 – 5.787) |
|  |  |  |  |
| Biopsy-Method * Lobar Location |  | 0.019 |  |
| CBCT-GB * RML^b^ | -2.507 | 0.030 | 0.081 (0.009 – 0.781) |
| CBCT-GB * RLL | -1.545 | 0.078 | 0.213 (0.038 – 1.192) |
| CBCT-GB * LUL^b^ | -4.004 | 0.002 | 0.018 (0.001 – 0.229) |
| CBCT-GB * Lingula | -1.853 | 0.069 | 0.157 (0.021 – 1.154) |
| CBCT-GB * LLL^b^ | -2.510 | 0.004 | 0.081 (0.015 – 0.450) |
|  |  |  |  |
| PPL Density |  | <0.001 |  |
| Solid (reference) | 0 (ref) |  | 1 (ref) |
| Part-Solid | -0.503 | 0.142 | 0.605 (0.309 – 1.184) |
| Ground Glass | -1.719 | <0.001 | 0.179 (0.076 – 0.424) |
|  |  |  |  |
| PPL Longest Diameter |  | 0.670 |  |
| <10mm (reference) | 0 (ref) |  | 1 (ref) |
| 10-20mm | 0.504 | 0.223 | 1.655 (0.735 – 3.726) |
| 20-30mm | 0.464 | 0.305 | 1.590 (0.656 – 3.854) |
| >=30mm | 0.400 | 0.416 | 1.492 (0.569 – 3.915) |
|  |  |  |  |
| PPL Peripheral Distribution |  | 0.297 |  |
| Inner One-Third (reference) | 0 (ref) |  | 1 (ref) |
| Middle One-Third | 0.612 | 0.200 | 1.844 (0.723 – 4.701) |
| Outer One-Third | 0.102 | 0.798 | 1.108 (0.505 – 2.428) |
|  |  |  |  |
| Bronchus Sign |  |  |  |
| Absent (reference) | 0 (ref) |  | 1 (ref) |
| Present | 0.868 | 0.032 | 2.381 (1.079 – 5.253) |

CI: Confidence Interval; PPL: Peripheral Pulmonary Lesion; CBCT-GB: Cone Beam Computed Tomography-Guided Bronchoscopy; CT-TTNB: Computed Tomography Guided TransThoracic Needle Biopsy; RUL: Right Upper Lobe; RML: Right Middle Lobe; RLL: Right Lower Lobe; LUL: Left Upper Lobe; LLL: Left Lower Lobe

^a^Nagelkerke R square = 13.9%. Hosmer and Lemeshow Test with p = 0.966. Classification Accuracy 92.0%.

^b^Statistically significant interactions were found for PPLs in the right middle lobe, left upper lobe, and left lower lobe.

**e-Table 4: Probability of Lobe-Specific Diagnostic Success after adjusting for PPL density, size, peripheral location, and bronchus sign (Calculated using parameter estimates and the model-based covariance matrix)**

| PPL Lobar Location^a^ | Adjusted Odds Ratio (95% CI) | *P*-Value |
| --- | --- | --- |
|  |  |  |
| RUL | 3.179 (0.836 – 12.092) | 0.090 |
| RML | 0.259 (0.041 – 1.648) | 0.152 |
| RLL | 0.678 (0.218 – 2.110) | 0.502 |
| LUL | 0.058 (0.007 – 0.513) | 0.010 |
| Lingula | 0.498 (0.110 – 2.263) | 0.367 |
| LLL | 0.258 (0.086 – 0.776) | 0.016 |

CI: Confidence Interval; PPL: Peripheral Pulmonary Lesion; CBCT-GB: Cone Beam Computed Tomography-Guided Bronchoscopy; CT-TTNB: Computed Tomography Guided TransThoracic Needle Biopsy; RUL: Right Upper Lobe; RML: Right Middle Lobe; RLL: Right Lower Lobe; LUL: Left Upper Lobe; LLL: Left Lower Lobe

^a^Probability of Diagnostic Success at 24 month follow up stratified by PPL lobar location after adjusting for PPL density, size, peripheral location, and bronchus sign. Shown are the odds ratios of CBCT-GB Group relative to CT-TTNB Group giving a diagnostic result per PPL lobar location.

**Non-Robotic CBCT-GB, Robotic CBCT-GB, CT-TTNB**

**Subgroup Comparisons**

**e-Table 5. Patient and Nodule Characteristics (Non-Robotic CBCT-GB, Robotic CBCT-GB, CT-TTNB)**

| Characteristic | Non-Robotic CBCT-GB  (N = 113) | Robotic CBCT-GB  (N = 271) | CT-TTNB  (N = 503) | *P-*Value |
| --- | --- | --- | --- | --- |
| Patients^a^ |  |  |  |  |
| Sex – No. (%) |  |  |  | 0.550 |
| Male | 62 (54.9) | 132 (48.7) | 256 (51.1) |  |
| Female | 51 (45.1) | 139 (51.3) | 245 (48.9) |  |
| Ethnicity – No. (%) |  |  |  | 0.031^b^ |
| White, Non-Hispanic | 67 (59.3) | 145 (53.5) | 241 (48.1) |  |
| Asian | 33 (29.2) | 81 (29.9) | 145 (28.9) |  |
| Other | 13 (11.5) | 45 (16.6) | 115 (23.0) |  |
| Smoking Status – No. (%) |  |  |  | 0.018^c^ |
| Current/Former Smoker 64 (56.6) | | 149 (55.0) | 230 (45.9) |  |
| Never Smoked | 49 (43.4) | 122 (45.0) | 271 (54.1) |  |
| Cancer History – No. (%) |  |  |  | <0.001^d^ |
| NSCLC | 22 (19.5) | 42 (15.5) | 76 (14.9) |  |
| SCLC | 0 (0.0) | 1 (0.4) | 2 (0.4) |  |
| Extra-Thoracic | 50 (44.2) | 111 (41.0) | 333 (65.2) |  |
| None | 45 (39.8) | 124 (45.8) | 100 (20.0) |  |
| Median Age – yr. (IQR) | 70 (62.5 – 77.5) | 69 (61.5 – 76.5) | 69 (61 - 77) | 0.380 |
| Median BMI (IQR) | 25.2 (21.9 – 28.6) | 25.1 (21.8 – 28.4) | 24.8 (21.5 – 28.2) | 0.874 |
|  |  |  |  |  |
| Nodules |  |  |  |  |
| Density – No. (%) |  |  |  | <0.004^e^ |
| Solid | 90 (77.6) | 212 (76.8) | 410 (81.5) |  |
| Part-Solid | 22 (19.0) | 56 (20.3) | 60 (11.9) |  |
| Ground Glass | 4 (3.4) | 8 (2.9) | 33 (6.6) |  |
| Lobar Location – No. (%) |  |  |  | 0.011^f^ |
| Right Upper | 28 (24.1) | 86 (31.2) | 121 (24.1) |  |
| Right Middle | 10 (8.6) | 28 (10.1) | 33 (6.6) |  |
| Right Lower | 17 (14.7) | 55 (19.9) | 114 (22.7) |  |
| Left Upper | 23 (19.8) | 46 (16.7) | 79 (15.7) |  |
| Lingula | 4 (3.4) | 20 (7.2) | 38 (7.6) |  |
| Left Lower | 34 (29.3) | 41 (14.9) | 118 (23.5) |  |
| Longest Diameter – No. (%) |  |  |  | <0.006^g^ |
| <10mm | 3 (2.6) | 19 (6.9) | 58 (11.5) |  |
| 10-20mm | 59 (50.9) | 134 (48.6) | 192 (38.2) |  |
| 20-30mm | 26 (22.4) | 63 (22.8) | 124 (24.7) |  |
| >=30mm | 28 (24.1) | 60 (21.7) | 129 (25.6) |  |
| Peripheral Distribution – No. (%) | |  |  | 0.500 |
| Inner third of lung | 14 (12.1) | 37 (13.4) | 53 (10.5) |  |
| Middle third of lung | 37 (31.9) | 77 (27.9) | 133 (26.4) |  |
| Outer third of lung | 65 (56.0) | 162 (58.7) | 317 (63.0) |  |
| Bronchus Sign Present^h^ – No. (%) 46 (39.7) | | 111 (40.2) | 62 (12.3) | <0.001 |
| TIL Confirmed^i^ – No. (%) | 113 (100.0) | 249 (95.0) | 494 (98.2) | 0.006^j^ |

CBCT-GB: Cone Beam Computed Tomography-Guided Biopsy; CT-TTNB: Computed Tomography Guided Transthoracic Needle Biopsy; NSCLC: Non-small cell lung carcinoma; SCLC: Small cell lung carcinoma; IQR: Inter-quartile range; BMI: Body Mass Index (weight in kilograms divided by square of height in meters); TIL: Tool in Lesion. Categorial variables are reported as counts and percentages and compared using Fisher’s Exact Test with a Bonferroni correction for sub-analyses. Continuous variables are reported as medians with IQR and compared using either the Mann-Whitney U Test or Median Test, as appropriate.

^a^Patient demographic percents are based on N = 113 for non-robotic CBCT-GB group, N = 271 for robotic CBCT-GB group, and N = 501 for CT-TTNB in order to not duplicate information for patients who had more than one PPL biopsied during the same procedure.

^b^The only statistical difference found was between non-robotic CBCT-GB and CT-TTNB in the other ethnicity category.

^c^The statistical difference was driven by difference in smoking status between robotic CBCT-GB and CT-TTNB.

^d^The statistical difference was driven by differences in the Extra-Thoracic and None categories between only the robotic CBCT-GB and CT-TTNB groups.

^e^The statistical difference was driven by differences in the Part-Solid category between robotic CBCT-GB and CT-TTNB.

^f^The statistical difference was driven by differences between robotic CBCT-GB and CT-TTNB in the LLL category.

^g^There were statistical differences in nodules <10mm between non-robotic CBCT-GB and CT-TTNB, and in nodules 10-20mm between both non-robotic and robotic CBCT-GB and CT-TTNB.

^h^The bronchus sign is present when a bronchial airway is found to terminate in or extend through a lung nodule on CT.

^i^TIL is confirmed when the biopsy tool is seen to be within the lesion on an intra-operative CT image.

^j^The statistical difference was driven by comparing robotic CBCT-GB to non-robotic CBCT-GB, and robotic CBCT-GB to CT-TTNB.

**e-Table 6. Primary and Secondary Outcomes (Non-Robotic CBCT-GB, Robotic CBCT-GB, CT-TTNB)**

| Outcome | Non-Robotic CBCT-GB  (N = 116) | Robotic CBCT-GB  (N = 276) | CT-TTNB  (N = 503) | *P*-Value |
| --- | --- | --- | --- | --- |
| Primary Outcome – No./Total No. (%) |  |  |  |  |
| Diagnostic Accuracy at 24 month follow up^a^ | 102/111 (91.9) | 238/264 (90.2) | 440/475 (92.6) | 0.501 |
| Probability of Malignancy at 24 month follow up given Non-Specific Result on Index Pathology | 6/26 (23.1) | 20/50 (40.0) | 21/89 (23.6) | 0.097 |
| Lost to Follow Up | 5/116 (4.3) | 12/276 (4.3) | 28/503 (5.6) | 0.705 |
|  |  |  |  |  |
| Secondary Outcomes – No./Total No. (%) |  |  |  |  |
| DA at 12 month follow up^a^ | 104/113 (92.0) | 243/271 (89.7) | 443/479 (92.5) | 0.404 |
| DY per ATS-ACCP^a^ | 96/116 (82.8) | 229/276 (83.0) | 428/503 (85.1) | 0.674 |
|  | | | |  |
| Secondary Procedures – No./No. Total (%) | | | |  |
| Subsequent Invasive Diagnostic Procedure^b^ | 7/116 (6.0) | 20/276 (7.2) | 32/503 (6.4) | 0.863 |
| Patients who met criteria for invasive mediastinal lymph node staging (cEBUS) at time of index biopsy^c^ | 31/116 (26.7) | 80/276 (29.0) | 93/503 (18.5) | 0.002^d^ |
| Of these patients, number who received cEBUS | 28/31 (90.3) | 68/80 (85.0) | 13/93 (14.0) | <0.001^e^ |
| Of those that received cEBUS, number whose lymph nodes returned positive for malignancy | 1/28 (3.6) | 6/68 (8.8) | 5/13 (38.5) | 0.003^e^ |

DA: Diagnostic Accuracy; DY: Diagnostic Yield; ATS: American Thoracic Society; ACCP: American College of Chest Physicians; cEBUS: Convex Endobronchial Ultrasound; CBCT-GB: Cone Beam Computed Tomography-Guided Biopsy; CT-TTNB: Computed Tomography Guided Transthoracic Needle Biopsy

^a^See Table 1 for varying definitions of diagnostic accuracy and diagnostic yield.

^b^A subsequent invasive diagnostic procedure is any procedure that involved biopsy of the same PPL, another PPL, or cEBUS of mediastinal lymph nodes

^c^Patients suspected of having malignancy without extra-thoracic metastases who also have either a PPL in central two thirds of lung fields, PPL size >= 3cm, or have enlarged or FDG avid mediastinal or hilar lymph nodes.

^d^The statistical difference was driven by differences between non-robotic CBCT-GB to both robotic CBCT-GB and CT-TTNB.

^e^The statistical difference was driven by differences between non-robotic CBCT-GB and both robotic CBCT-GB to CT-TTNB.

**e-Table 7. Safety Outcomes (Non-Robotic CBCT-GB, Robotic CBCT-GB, CT-TTNB)**

| Complication^a^ – No. (%) | Non-Robotic CBCT-GB  (N = 116) | Robotic CBCT-GB  (N = 276) | CT-TTNB  (N = 503) | *P-*Value |
| --- | --- | --- | --- | --- |
| Any complication | 8 (6.9) | 9 (3.3) | 209 (41.6) | <0.001^b^ |
| Pneumothorax without Intervention^c^ | 1 (0.9) | 4 (1.4) | 130 (25.8) | <0.001^b^ |
| Pneumothorax with Intervention^c^ | 0 (0.0) | 2 (0.7) | 28 (5.6) | <0.001^b^ |
| Serious^d^ | 6 (5.2) | 7 (2.5) | 30 (6.0) | <0.001^b^ |
| Death | 1 (0.9) | 0 (0.0) | 0 (0.0) | 1.000 |

CBCT-GB: Cone Beam Computed Tomography-Guided Biopsy; CT-TTNB: Computed Tomography Guided Transthoracic Needle Biopsy;

^a^Individual complication numbers may surpass total number of complications, as a patient could have more than one type of complication.

^b^There was no statistical difference between Non-Robotic and Robotic CBCT-GB. However, both groups differed from CT-TTNB for all of these complication types.

^c^Intervention was defined as anything other than observation of the pneumothorax. Such Interventions included any of the following: needle aspiration, pigtail catheter placement, or surgical chest tube placement.

^d^Serious Complications were defined as any degree of bleeding requiring blood transfusion or hospital admission, respiratory failure necessitating the use of non-invasive or invasive ventilation, impending cardiac compromise requiring vasopressor support, or overnight hospital admission for any reason.

**e-Table 8. Univariate Analysis of Primary Outcome of Diagnostic Accuracy at 24 month follow up (Non-Robotic CBCT-GB, Robotic CBCT-GB, CT-TTNB)**

|  | Non-Robotic CBCT-GB | Robotic CBCT-GB | CT-TTNB | *P*-Value |
| --- | --- | --- | --- | --- |
|  | No. Patients/Total No. (%) | | |  |
| Ethnicity |  |  |  |  |
| White, Non-Hispanic | 63/65 (96.9) | 122/140 (87.1) | 211/225 (93.8) | 0.022 |
| Asian | 26/31 (83.9) | 72/78 (92.3) | 130/141 (92.2) | 0.306 |
| Other | 10/12 (83.3) | 41/42 (97.6) | 97/107 (90.7) | 0.196 |
| Smoking Status |  |  |  |  |
| Current/Former | 57/61 (93.4) | 129/142 (90.8) | 200/214 (93.5) | 0.629 |
| Never Smoked | 42/47 (89.4) | 106/118 (89.8) | 238/259 (91.9) | 0.739 |
| Prior Cancer History |  |  |  |  |
| Yes | 63/68 (92.6) | 127/147 (86.4) | 356/385 (92.5) | 0.080 |
| None | 39/43 (90.7) | 111/117 (94.9) | 84/90 (93.3) | 0.628 |
| PPL Density |  |  |  |  |
| Solid | 80/86 (93.0) | 185/204 (90.7) | 369/389 (94.9) | 0.151 |
| Part-Solid | 20/21 (95.2) | 47/53 (88.7) | 47/54 (87.0) | 0.589 |
| Ground Glass | 2/4 (50.0) | 6/7 (85.7) | 24/32 (75.0) | 0.422 |
| PPL Lobar Location |  |  |  |  |
| Right Upper | 28/28 (100.0) | 76/79 (96.2) | 102/114 (89.5) | 0.059 |
| Right Middle | 9/10 (90.0) | 25/28 (89.3) | 30/32 (93.8) | 0.815 |
| Right Lower | 17/17 (100.0) | 48/54 (88.9) | 99/108 (91.7) | 0.353 |
| Left Upper | 17/21 (81.0) | 41/45 (91.1) | 72/73 (98.6) | 0.011 |
| Lingula | 3/4 (75.0) | 16/19 (84.2) | 30/35 (85.7) | 0.854 |
| Left Lower | 28/31 (90.3) | 32/39 (82.1) | 107/113 (94.7) | 0.054 |
| PPL Longest Diameter |  |  |  |  |
| <10mm | 2/3 (66.7) | 12/16 (75.0) | 45/52 (86.5) | 0.414 |
| 10-20mm | 50/55 (90.9) | 113/126 (89.7) | 170/181 (93.9) | 0.384 |
| 20-30mm | 23/25 (92.0) | 57/62 (91.9) | 109/118 (92.4) | 0.994 |
| >=30mm | 27/28 (96.4) | 56/60 (93.3) | 116/124 (93.5) | 0.831 |
| PPL Distribution |  |  |  |  |
| Inner third of lung | 12/14 (85.7) | 29/34 (85.3) | 48/50 (96.0) | 0.193 |
| Middle third of lung | 35/35 (100.0) | 70/75 (93.3) | 118/125 (94.4) | 0.313 |
| Outer third of lung | 55/62 (88.7) | 139/155 (89.7) | 274/300 (91.3) | 0.742 |
| Bronchus Sign^a^ |  |  |  |  |
| Present | 43/45 (95.6) | 105/109 (96.3) | 57/61 (93.4) | 0.690 |
| Absent | 59/66 (89.4) | 133/155 (85.8) | 383/414 (92.5) | 0.049 |
|  |  |  |  |  |

PPL: Peripheral Pulmonary Lesion; CBCT-GB: Cone Beam Computed Tomography Guided Bronchoscopy; CT-TTNB: Computed Tomography Guided TransThoracic Needle Biopsy

^a^The bronchus sign is present when a bronchial airway is found to terminate in or extend through a lung nodule on CT.

**HIGH ORDER CONTINGENCY TABLES**

**e-Table 9. High Order Contingency Tables for Primary Outcome of Diagnostic Accuracy at 24 months of follow up (Non-Robotic CBCT-GB versus Robotic CBCT-GB)^a^**

| **Non-Robotic Cone Beam Computed Tomography Guided Biopsy** | | **Reference Standard of Truth Result** | | | | | | | | | | | |
| --- | --- | --- | --- | --- | --- | --- | --- | --- | --- | --- | --- | --- | --- |
|  |  | NSCLC | SCLC | Pulmonary Carcinoid | Metastases to Lung | Infection | Benign Nodule | Sarcoid | Stable >=24 months | Shrank or Resolved | Enlarged | <24 months follow up | Total |
| ***Varies^b^***  ***Non-Diagnostic***  ***Diagnostic***  **Index Pathology Result** | NSCLC | 54 | 0 | 0 | 0 | 0 | 0 | 0 | 0 | 0 | 0 | 0 | 54 |
|  | SCLC | 0 | 0 | 0 | 0 | 0 | 0 | 0 | 0 | 0 | 0 | 0 | 0 |
|  | Pulmonary Carcinoid | 0 | 0 | 3 | 0 | 0 | 0 | 0 | 0 | 0 | 0 | 0 | 3 |
|  | Metastases to Lung | 0 | 0 | 0 | 14 | 0 | 0 | 0 | 0 | 0 | 0 | 0 | 14 |
|  | Infection | 0 | 0 | 0 | 0 | 10 | 0 | 0 | 0 | 0 | 0 | 0 | 10 |
|  | Benign Nodule | 0 | 0 | 0 | 0 | 0 | 4 | 0 | 0 | 0 | 0 | 0 | 4 |
|  | Organizing Pneumonia^b^ | 0 | 0 | 0 | 0 | 0 | 0 | 0 | 1 | 4 | 0 | 2 | 7 |
|  | Granulomatous Inflammation^b^ | 0 | 0 | 0 | 0 | 0 | 0 | 0 | 1 | 1 | 0 | 2 | 4 |
|  | Fibroelastotic Scar | 0 | 0 | 0 | 0 | 0 | 0 | 0 | 5 | 1 | 0 | 0 | 6 |
|  | Necrosis | 0 | 0 | 0 | 0 | 0 | 0 | 0 | 0 | 0 | 0 | 0 | 0 |
|  | Non-Specific Inflammation | 0 | 0 | 0 | 1 | 0 | 0 | 0 | 2 | 2 | 0 | 0 | 5 |
|  | Atypical Cells | 2 | 0 | 0 | 1 | 0 | 0 | 0 | 0 | 0 | 0 | 1 | 4 |
|  | Non-Specific Normal Lung Tissue | 2 | 0 | 0 | 0 | 0 | 0 | 0 | 3 | 0 | 0 | 0 | 5 |
|  | Total | 58 | 0 | 3 | 16 | 10 | 4 | 0 | 12 | 8 | 0 | 5 | 116 |
| **Robotic Computed Tomography Guided TransThoracic Needle Biopsy** | | **Reference Standard of Truth Result** | | | | | | | | | | | |
|  |  | NSCLC | SCLC | Pulmonary Carcinoid | Metastases to Lung | Infection | Benign Nodule | Sarcoid | Stable >=24 months | Shrank or Resolved | Enlarged | <24 months follow up | Total |
|  | NSCLC | 123 | 0 | 0 | 0 | 0 | 0 | 0 | 0 | 0 | 0 | 0 | 123 |
|  | SCLC | 0 | 0 | 0 | 0 | 0 | 0 | 0 | 0 | 0 | 0 | 0 | 0 |
|  | Pulmonary Carcinoid | 0 | 0 | 6 | 0 | 0 | 0 | 0 | 0 | 0 | 0 | 0 | 6 |
|  | Metastases to Lung  ***Varies^b^***  ***Non-Diagnostic***  ***Diagnostic***  **Index Pathology Result** | 0 | 0 | 0 | 40 | 0 | 0 | 0 | 0 | 0 | 0 | 0 | 40 |
|  | Infection | 0 | 0 | 0 | 0 | 38 | 0 | 0 | 0 | 0 | 0 | 0 | 38 |
|  | Benign Nodule | 0 | 0 | 0 | 0 | 0 | 5 | 0 | 0 | 0 | 0 | 0 | 5 |
|  | Organizing Pneumonia^b^ | 1 | 0 | 0 | 1 | 0 | 0 | 0 | 1 | 8 | 0 | 1 | 12 |
|  | Granulomatous Inflammation^b^ | 0 | 0 | 0 | 0 | 0 | 0 | 2 | 1 | 1 | 0 | 1 | 5 |
|  | Fibroelastotic Scar | 2 | 0 | 0 | 0 | 0 | 0 | 0 | 4 | 3 | 0 | 4 | 13 |
|  | Necrosis | 0 | 0 | 0 | 2 | 1 | 0 | 0 | 0 | 0 | 0 | 0 | 3 |
|  | Non-Specific Inflammation | 0 | 0 | 0 | 0 | 2 | 1 | 0 | 0 | 2 | 1 | 2 | 8 |
|  | Atypical Cells | 6 | 0 | 0 | 1 | 0 | 0 | 0 | 0 | 0 | 0 | 0 | 7 |
|  | Non-Specific Normal Lung Tissue | 2 | 0 | 1 | 4 | 0 | 0 | 0 | 3 | 1 | 1 | 4 | 16 |
|  | Total | 134 | 0 | 7 | 48 | 41 | 6 | 2 | 9 | 15 | 2 | 12 | 276 |

NSCLC: Non-small cell lung carcinoma; SCLC: Small cell lung carcinoma; CBCT-GB: Cone Beam Computed Tomography Guided Bronchoscopy; CT-TTNB: Computed Tomography Guided TransThoracic Needle Biopsy; ATS-ACCP: American Thoracic Society-American College of Chest Physicians; DY: Diagnostic Yield; DA: Diagnostic Accuracy.

^a^This table shows into what category every index pathology result was put into after 24 months of follow up data. For further clarity on definitions of terms, see Table 1.

^b^Organizing Pneumonia and Granulomatous Inflammation are considered diagnostic on index pathology per the ATS-ACCP definition of DY^26^. Both are considered non-diagnostic on index pathology per the definitions of DA employed in this paper.

**e-Table 10. High Order Contingency Tables for classification of infection type after 24 months of follow up (CBCT-GB versus CT-TTNB)^a^**

| **Cone Beam Computed Tomography Guided Biopsy** | | **Reference Standard of Truth Result** | | | | | | |
| --- | --- | --- | --- | --- | --- | --- | --- | --- |
|  |  | Tuberculosis | Non-tuberculosis Mycobacteria | Aspergillus | Coccidioidomycosis | Other Fungal | Other | Total |
| **Index Pathology Result** | Tuberculosis | 4 | 0 | 0 | 0 | 0 | 0 | 4 |
|  | Non-tuberculosis Mycobacteria | 0 | 5 | 0 | 0 | 0 | 0 | 5 |
|  | Aspergillus | 0 | 0 | 9 | 0 | 0 | 0 | 9 |
|  | Coccidiodomycosis | 0 | 0 | 0 | 5 | 0 | 0 | 5 |
|  | Other Fungal | 0 | 0 | 0 | 0 | 10 | 0 | 10 |
|  | Other | 0 | 0 | 0 | 0 | 0 | 15 | 15 |
|  | Total | 4 | 5 | 9 | 5 | 10 | 15 | 48 |
| **Computed Tomography Guided TransThoracic Needle Biopsy** | | **Reference Standard of Truth Result** | | | | | | |
|  |  | Tuberculosis | Non-tuberculosis Mycobacteria | Aspergillus | Coccidioidomycosis | Other Fungal | Other | Total |
| **Index Pathology Result** | Tuberculosis | 1 | 0 | 0 | 0 | 0 | 0 | 1 |
|  | Non-tuberculosis Mycobacteria | 0 | 3 | 0 | 0 | 0 | 0 | 3 |
|  | Aspergillus | 0 | 0 | 2 | 0 | 0 | 0 | 2 |
|  | Coccidiodomycosis | 0 | 0 | 0 | 5 | 0 | 0 | 5 |
|  | Other Fungal | 0 | 0 | 0 | 0 | 9 | 0 | 9 |
|  | Other | 0 | 0 | 0 | 0 | 0 | 6 | 6 |
|  | Total | 1 | 3 | 2 | 5 | 9 | 6 | 26 |
| **Any Biopsy Method**  **(CBCT-GB or CT-TTNB)** | | **Reference Standard of Truth Result** | | | | | | |
|  |  | Tuberculosis | Non-tuberculosis Mycobacteria | Aspergillus | Coccidioidomycosis | Other Fungal | Other | Total |
| **Index Pathology Result** | Tuberculosis | 5 | 0 | 0 | 0 | 0 | 0 | 5 |
|  | Non-tuberculosis Mycobacteria | 0 | 8 | 0 | 0 | 0 | 0 | 8 |
|  | Aspergillus | 0 | 0 | 11 | 0 | 0 | 0 | 11 |
|  | Coccidiodomycosis | 0 | 0 | 0 | 10 | 0 | 0 | 10 |
|  | Other Fungal | 0 | 0 | 0 | 0 | 19 | 0 | 19 |
|  | Other | 0 | 0 | 0 | 0 | 0 | 21 | 21 |
|  | Total | 5 | 8 | 11 | 10 | 19 | 21 | 74 |

NSCLC: Non-small cell lung carcinoma; SCLC: Small cell lung carcinoma; CBCT-GB: Cone Beam Computed Tomography Guided Bronchoscopy; CT-TTNB: Computed Tomography Guided TransThoracic Needle Biopsy.

^a^This table shows into what category every index infection result was put into after 24 months of follow up data.

**e-Table 11. High Order Contingency Tables for classification of infection type after 24 months of follow up (Non-Robotic CBCT-GB versus Robotic CBCT-GB)^a^**

| **Non-Robotic Cone Beam Computed Tomography Guided Biopsy** | | **Reference Standard of Truth Result** | | | | | | |
| --- | --- | --- | --- | --- | --- | --- | --- | --- |
|  |  | Tuberculosis | Non-tuberculosis Mycobacteria | Aspergillus | Coccidioidomycosis | Other Fungal | Other | Total |
| **Index Pathology Result** | Tuberculosis | 0 | 0 | 0 | 0 | 0 | 0 | 0 |
|  | Non-tuberculosis Mycobacteria | 0 | 0 | 0 | 0 | 0 | 0 | 0 |
|  | Aspergillus | 0 | 0 | 1 | 0 | 0 | 0 | 1 |
|  | Coccidiodomycosis | 0 | 0 | 0 | 0 | 0 | 0 | 2 |
|  | Other Fungal | 0 | 0 | 0 | 0 | 2 | 0 | 0 |
|  | Other | 0 | 0 | 0 | 0 | 0 | 7 | 7 |
|  | Total | 0 | 0 | 1 | 0 | 2 | 7 | 10 |
| **Robotic Computed Tomography Guided TransThoracic Needle Biopsy** | | **Reference Standard of Truth Result** | | | | | | |
|  |  | Tuberculosis | Non-tuberculosis Mycobacteria | Aspergillus | Coccidioidomycosis | Other Fungal | Other | Total |
| **Index Pathology Result** | Tuberculosis | 4 | 0 | 0 | 0 | 0 | 0 | 4 |
|  | Non-tuberculosis Mycobacteria | 0 | 5 | 0 | 0 | 0 | 0 | 5 |
|  | Aspergillus | 0 | 0 | 8 | 0 | 0 | 0 | 8 |
|  | Coccidiodomycosis | 0 | 0 | 0 | 5 | 0 | 0 | 5 |
|  | Other Fungal | 0 | 0 | 0 | 0 | 8 | 0 | 8 |
|  | Other | 0 | 0 | 0 | 0 | 0 | 8 | 8 |
|  | Total | 4 | 5 | 8 | 5 | 8 | 8 | 38 |

NSCLC: Non-small cell lung carcinoma; SCLC: Small cell lung carcinoma; CBCT-GB: Cone Beam Computed Tomography Guided Bronchoscopy; CT-TTNB: Computed Tomography Guided TransThoracic Needle Biopsy.

^a^This table shows into what category every index infection result was put into after 24 months of follow up data.

**e-Table 12. High Order Contingency Tables for Secondary Outcome of Diagnostic Accuracy at 12 months of follow up^a^**

| **Cone Beam Computed Tomography Guided Biopsy** | | **Reference Standard of Truth Result** | | | | | | | | | | | |
| --- | --- | --- | --- | --- | --- | --- | --- | --- | --- | --- | --- | --- | --- |
|  |  | NSCLC | SCLC | Pulmonary Carcinoid | Metastases to Lung | Infection | Benign Nodule | Sarcoid | Stable >=12 months | Shrank or Resolved | Enlarged | <12 months follow up | Total |
| **Index Pathology Result**  ***Diagnostic***  ***Non-Diagnostic***  ***Varies^b^*** | NSCLC | 177 | 0 | 0 | 0 | 0 | 0 | 0 | 0 | 0 | 0 | 0 | 177 |
|  | SCLC | 0 | 0 | 0 | 0 | 0 | 0 | 0 | 0 | 0 | 0 | 0 | 0 |
|  | Pulmonary Carcinoid | 0 | 0 | 9 | 0 | 0 | 0 | 0 | 0 | 0 | 0 | 0 | 9 |
|  | Metastases to Lung | 0 | 0 | 0 | 54 | 0 | 0 | 0 | 0 | 0 | 0 | 0 | 54 |
|  | Infection | 0 | 0 | 0 | 0 | 48 | 0 | 0 | 0 | 0 | 0 | 0 | 48 |
|  | Benign Nodule | 0 | 0 | 0 | 0 | 0 | 9 | 0 | 0 | 0 | 0 | 0 | 9 |
|  | Organizing Pneumonia^b^ | 1 | 0 | 0 | 1 | 0 | 0 | 0 | 3 | 12 | 0 | 2 | 19 |
|  | Granulomatous Inflammation^b^ | 0 | 0 | 0 | 0 | 0 | 0 | 2 | 4 | 2 | 0 | 1 | 9 |
|  | Fibroelastotic Scar | 2 | 0 | 0 | 0 | 0 | 0 | 0 | 11 | 4 | 0 | 2 | 19 |
|  | Necrosis | 0 | 0 | 0 | 2 | 1 | 0 | 0 | 0 | 0 | 0 | 0 | 3 |
|  | Non-Specific Inflammation | 0 | 0 | 0 | 1 | 2 | 1 | 0 | 4 | 4 | 1 | 0 | 13 |
|  | Atypical Cells | 8 | 0 | 0 | 2 | 0 | 0 | 0 | 0 | 0 | 0 | 1 | 11 |
|  | Non-Specific Normal Lung Tissue | 4 | 0 | 1 | 4 | 0 | 0 | 0 | 8 | 1 | 1 | 2 | 21 |
|  | Total | 192 | 0 | 10 | 64 | 51 | 10 | 2 | 30 | 23 | 2 | 8 | 392 |
| **Computed Tomography Guided TransThoracic Needle Biopsy** | | **Reference Standard of Truth Result** | | | | | | | | | | | |
|  |  | NSCLC | SCLC | Pulmonary Carcinoid | Metastases to Lung | Infection | Benign Nodule | Sarcoid | Stable >=12 months | Shrank or Resolved | Enlarged | <12 months follow up | Total |
|  | NSCLC | 131 | 0 | 0 | 0 | 0 | 0 | 0 | 0 | 0 | 0 | 0 | 131 |
|  | SCLC | 0 | 7 | 0 | 0 | 0 | 0 | 0 | 0 | 0 | 0 | 0 | 7 |
|  | Pulmonary Carcinoid | 0 | 0 | 1 | 0 | 0 | 0 | 0 | 0 | 0 | 0 | 0 | 1 |
|  | Metastases to Lung  **Index Pathology Result**  ***Diagnostic***  ***Non-Diagnostic***  ***Varies^b^*** | 0 | 0 | 0 | 211 | 0 | 0 | 0 | 0 | 0 | 0 | 0 | 211 |
|  | Infection | 0 | 0 | 0 | 0 | 26 | 0 | 0 | 0 | 0 | 0 | 0 | 26 |
|  | Benign Nodule | 0 | 0 | 0 | 0 | 0 | 6 | 0 | 0 | 0 | 0 | 0 | 6 |
|  | Organizing Pneumonia^b^ | 0 | 0 | 0 | 2 | 4 | 0 | 0 | 3 | 8 | 2 | 5 | 24 |
|  | Granulomatous Inflammation^b^ | 1 | 0 | 0 | 0 | 0 | 0 | 2 | 8 | 7 | 0 | 4 | 22 |
|  | Fibroelastotic Scar | 1 | 0 | 0 | 2 | 0 | 0 | 0 | 6 | 1 | 0 | 2 | 12 |
|  | Necrosis | 0 | 0 | 0 | 2 | 2 | 1 | 0 | 0 | 2 | 1 | 0 | 8 |
|  | Non-Specific Inflammation | 0 | 0 | 0 | 1 | 3 | 0 | 0 | 5 | 9 | 0 | 8 | 26 |
|  | Atypical Cells | 9 | 0 | 0 | 1 | 1 | 0 | 0 | 3 | 1 | 0 | 2 | 17 |
|  | Non-Specific Normal Lung Tissue | 0 | 0 | 0 | 2 | 1 | 1 | 0 | 2 | 2 | 1 | 3 | 12 |
|  | Total | 142 | 7 | 1 | 221 | 37 | 8 | 2 | 27 | 30 | 4 | 24 | 503 |
| **Any Biopsy Method (CBCT-GB or CT-TTNB)** | | **Reference Standard of Truth Result** | | | | | | | | | | | |
|  |  | NSCLC | SCLC | Pulmonary Carcinoid | Metastases to Lung | Infection | Benign Nodule | Sarcoid | Stable >=12 months | Shrank or Resolved | Enlarged | <12 months follow up | Total |
|  | NSCLC | 308 | 0 | 0 | 0 | 0 | 0 | 0 | 0 | 0 | 0 | 0 | 308 |
|  | SCLC | 0 | 7 | 0 | 0 | 0 | 0 | 0 | 0 | 0 | 0 | 0 | 7 |
|  | Pulmonary Carcinoid | 0 | 0 | 10 | 0 | 0 | 0 | 0 | 0 | 0 | 0 | 0 | 10 |
|  | Metastases to Lung  **Index Pathology Result**  ***Diagnostic***  ***Non-Diagnostic***  ***Varies^b^*** | 0 | 0 | 0 | 265 | 0 | 0 | 0 | 0 | 0 | 0 | 0 | 265 |
|  | Infection | 0 | 0 | 0 | 0 | 74 | 0 | 0 | 0 | 0 | 0 | 0 | 74 |
|  | Benign Nodule | 0 | 0 | 0 | 0 | 0 | 15 | 0 | 0 | 0 | 0 | 0 | 15 |
|  | Organizing Pneumonia^b^ | 1 | 0 | 0 | 3 | 4 | 0 | 0 | 6 | 20 | 2 | 7 | 43 |
|  | Granulomatous Inflammation^b^ | 1 | 0 | 0 | 0 | 0 | 0 | 4 | 12 | 9 | 0 | 5 | 31 |
|  | Fibroelastotic Scar | 3 | 0 | 0 | 2 | 0 | 0 | 0 | 17 | 5 | 0 | 4 | 31 |
|  | Necrosis | 0 | 0 | 0 | 4 | 3 | 1 | 0 | 0 | 2 | 1 | 0 | 11 |
|  | Non-Specific Inflammation | 0 | 0 | 0 | 2 | 5 | 1 | 0 | 9 | 13 | 1 | 8 | 39 |
|  | Atypical Cells | 17 | 0 | 0 | 3 | 1 | 0 | 0 | 3 | 1 | 0 | 3 | 28 |
|  | Non-Specific Normal Lung Tissue | 4 | 0 | 1 | 6 | 1 | 1 | 0 | 10 | 3 | 2 | 5 | 33 |
|  | Total | 334 | 7 | 11 | 285 | 88 | 18 | 4 | 57 | 53 | 6 | 32 | 895 |

NSCLC: Non-small cell lung carcinoma; SCLC: Small cell lung carcinoma; CBCT-GB: Cone Beam Computed Tomography Guided Bronchoscopy; CT-TTNB: Computed Tomography Guided TransThoracic Needle Biopsy; ATS-ACCP: American Thoracic Society-American College of Chest Physicians; DY: Diagnostic Yield; DA: Diagnostic Accuracy.

^a^This table shows into what category every index pathology result was put into after 24 months of follow up data. For further clarity on definitions of terms, see Table 1.

^b^Organizing Pneumonia and Granulomatous Inflammation are considered diagnostic on index pathology per the ATS-ACCP definition of DY^26^. Both are considered non-diagnostic on index pathology per the definitions of DA employed in this paper.

**e-Table 13. High Order Contingency Tables for Secondary Outcome of Diagnostic Accuracy at 12 months of follow up (Non-Robotic CBCT-GB versus Robotic CBCT-GB)^a^**

| **Non-Robotic Cone Beam Computed Tomography Guided Biopsy** | | **Reference Standard of Truth Result** | | | | | | | | | | | |
| --- | --- | --- | --- | --- | --- | --- | --- | --- | --- | --- | --- | --- | --- |
|  |  | NSCLC | SCLC | Pulmonary Carcinoid | Metastases to Lung | Infection | Benign Nodule | Sarcoid | Stable >=12 months | Shrank or Resolved | Enlarged | <12 months follow up | Total |
|  | NSCLC | 54 | 0 | 0 | 0 | 0 | 0 | 0 | 0 | 0 | 0 | 0 | 54 |
|  | SCLC | 0 | 0 | 0 | 0 | 0 | 0 | 0 | 0 | 0 | 0 | 0 | 0 |
|  | Pulmonary Carcinoid | 0 | 0 | 3 | 0 | 0 | 0 | 0 | 0 | 0 | 0 | 0 | 3 |
|  | Metastases to Lung  **Index Pathology Result**  ***Diagnostic***  ***Non-Diagnostic***  ***Varies^b^*** | 0 | 0 | 0 | 14 | 0 | 0 | 0 | 0 | 0 | 0 | 0 | 14 |
|  | Infection | 0 | 0 | 0 | 0 | 10 | 0 | 0 | 0 | 0 | 0 | 0 | 10 |
|  | Benign Nodule | 0 | 0 | 0 | 0 | 0 | 4 | 0 | 0 | 0 | 0 | 0 | 4 |
|  | Organizing Pneumonia^b^ | 0 | 0 | 0 | 0 | 0 | 0 | 0 | 2 | 4 | 0 | 1 | 7 |
|  | Granulomatous Inflammation^b^ | 0 | 0 | 0 | 0 | 0 | 0 | 0 | 2 | 1 | 0 | 1 | 4 |
|  | Fibroelastotic Scar | 0 | 0 | 0 | 0 | 0 | 0 | 0 | 5 | 1 | 0 | 0 | 6 |
|  | Necrosis | 0 | 0 | 0 | 0 | 0 | 0 | 0 | 0 | 0 | 0 | 0 | 0 |
|  | Non-Specific Inflammation | 0 | 0 | 0 | 1 | 0 | 0 | 0 | 2 | 2 | 0 | 0 | 5 |
|  | Atypical Cells | 2 | 0 | 0 | 1 | 0 | 0 | 0 | 0 | 0 | 0 | 1 | 4 |
|  | Non-Specific Normal Lung Tissue | 2 | 0 | 0 | 0 | 0 | 0 | 0 | 3 | 0 | 0 | 0 | 5 |
|  | Total | 58 | 0 | 3 | 16 | 10 | 4 | 0 | 14 | 8 | 0 | 3 | 116 |
| **Robotic Computed Tomography Guided TransThoracic Needle Biopsy** | | **Reference Standard of Truth Result** | | | | | | | | | | | |
|  |  | NSCLC | SCLC | Pulmonary Carcinoid | Metastases to Lung | Infection | Benign Nodule | Sarcoid | Stable >=12 months | Shrank or Resolved | Enlarged | <12 months follow up | Total |
|  | NSCLC | 123 | 0 | 0 | 0 | 0 | 0 | 0 | 0 | 0 | 0 | 0 | 123 |
|  | SCLC | 0 | 0 | 0 | 0 | 0 | 0 | 0 | 0 | 0 | 0 | 0 | 0 |
|  | Pulmonary Carcinoid  **Index Pathology Result**  ***Diagnostic***  ***Non-Diagnostic***  ***Varies^b^*** | 0 | 0 | 6 | 0 | 0 | 0 | 0 | 0 | 0 | 0 | 0 | 6 |
|  | Metastases to Lung | 0 | 0 | 0 | 40 | 0 | 0 | 0 | 0 | 0 | 0 | 0 | 40 |
|  | Infection | 0 | 0 | 0 | 0 | 38 | 0 | 0 | 0 | 0 | 0 | 0 | 38 |
|  | Benign Nodule | 0 | 0 | 0 | 0 | 0 | 5 | 0 | 0 | 0 | 0 | 0 | 5 |
|  | Organizing Pneumonia^b^ | 1 | 0 | 0 | 1 | 0 | 0 | 0 | 1 | 8 | 0 | 1 | 12 |
|  | Granulomatous Inflammation^b^ | 0 | 0 | 0 | 0 | 0 | 0 | 2 | 2 | 1 | 0 | 0 | 5 |
|  | Fibroelastotic Scar | 2 | 0 | 0 | 0 | 0 | 0 | 0 | 6 | 3 | 0 | 2 | 13 |
|  | Necrosis | 0 | 0 | 0 | 2 | 1 | 0 | 0 | 0 | 0 | 0 | 0 | 3 |
|  | Non-Specific Inflammation | 0 | 0 | 0 | 0 | 2 | 1 | 0 | 2 | 2 | 1 | 0 | 8 |
|  | Atypical Cells | 6 | 0 | 0 | 1 | 0 | 0 | 0 | 0 | 0 | 0 | 0 | 7 |
|  | Non-Specific Normal Lung Tissue | 2 | 0 | 1 | 4 | 0 | 0 | 0 | 5 | 1 | 1 | 2 | 16 |
|  | Total | 134 | 0 | 7 | 48 | 41 | 6 | 2 | 16 | 15 | 2 | 5 | 276 |

NSCLC: Non-small cell lung carcinoma; SCLC: Small cell lung carcinoma; CBCT-GB: Cone Beam Computed Tomography Guided Bronchoscopy; CT-TTNB: Computed Tomography Guided TransThoracic Needle Biopsy; ATS-ACCP: American Thoracic Society-American College of Chest Physicians; DY: Diagnostic Yield; DA: Diagnostic Accuracy.

^a^This table shows into what category every index pathology result was put into after 24 months of follow up data. For further clarity on definitions of terms, see Table 1.

^b^Organizing Pneumonia and Granulomatous Inflammation are considered diagnostic on index pathology per the ATS-ACCP definition of DY^26^. Both are considered non-diagnostic on index pathology per the definitions of DA employed in this paper.

**e-Table 14. High Order Contingency Tables for classification of infection type after 12 months of follow up (CBCT-GB versus CT-TTNB)^a^**

| **Cone Beam Computed Tomography Guided Biopsy** | | **Reference Standard of Truth Result** | | | | | | |
| --- | --- | --- | --- | --- | --- | --- | --- | --- |
|  |  | Tuberculosis | Non-tuberculosis Mycobacteria | Aspergillus | Coccidioidomycosis | Other Fungal | Other | Total |
| **Index Pathology Result** | Tuberculosis | 4 | 0 | 0 | 0 | 0 | 0 | 4 |
|  | Non-tuberculosis Mycobacteria | 0 | 5 | 0 | 0 | 0 | 0 | 5 |
|  | Aspergillus | 0 | 0 | 9 | 0 | 0 | 0 | 9 |
|  | Coccidiodomycosis | 0 | 0 | 0 | 5 | 0 | 0 | 5 |
|  | Other Fungal | 0 | 0 | 0 | 0 | 10 | 0 | 10 |
|  | Other | 0 | 0 | 0 | 0 | 0 | 15 | 15 |
|  | Total | 4 | 5 | 9 | 5 | 10 | 15 | 48 |
| **Computed Tomography Guided TransThoracic Needle Biopsy** | | **Reference Standard of Truth Result** | | | | | | |
|  |  | Tuberculosis | Non-tuberculosis Mycobacteria | Aspergillus | Coccidioidomycosis | Other Fungal | Other | Total |
| **Index Pathology Result** | Tuberculosis | 1 | 0 | 0 | 0 | 0 | 0 | 1 |
|  | Non-tuberculosis Mycobacteria | 0 | 3 | 0 | 0 | 0 | 0 | 3 |
|  | Aspergillus | 0 | 0 | 2 | 0 | 0 | 0 | 2 |
|  | Coccidiodomycosis | 0 | 0 | 0 | 5 | 0 | 0 | 5 |
|  | Other Fungal | 0 | 0 | 0 | 0 | 9 | 0 | 9 |
|  | Other | 0 | 0 | 0 | 0 | 0 | 6 | 6 |
|  | Total | 1 | 3 | 2 | 5 | 9 | 6 | 26 |
| **Any Biopsy Method**  **(CBCT-GB or CT-TTNB)** | | **Reference Standard of Truth Result** | | | | | | |
|  |  | Tuberculosis | Non-tuberculosis Mycobacteria | Aspergillus | Coccidioidomycosis | Other Fungal | Other | Total |
| **Index Pathology Result** | Tuberculosis | 5 | 0 | 0 | 0 | 0 | 0 | 5 |
|  | Non-tuberculosis Mycobacteria | 0 | 8 | 0 | 0 | 0 | 0 | 8 |
|  | Aspergillus | 0 | 0 | 11 | 0 | 0 | 0 | 11 |
|  | Coccidiodomycosis | 0 | 0 | 0 | 10 | 0 | 0 | 10 |
|  | Other Fungal | 0 | 0 | 0 | 0 | 19 | 0 | 19 |
|  | Other | 0 | 0 | 0 | 0 | 0 | 21 | 21 |
|  | Total | 5 | 8 | 11 | 10 | 19 | 21 | 74 |

NSCLC: Non-small cell lung carcinoma; SCLC: Small cell lung carcinoma; CBCT-GB: Cone Beam Computed Tomography Guided Bronchoscopy; CT-TTNB: Computed Tomography Guided TransThoracic Needle Biopsy.

^a^This table shows into what category every index infection result was put into after 12 months of follow up data.

**e-Table 15. High Order Contingency Tables for classification of infection type after 12 months of follow up (Non-Robotic CBCT-GB versus Robotic CBCT-GB)^a^**

| **Non-Robotic Cone Beam Computed Tomography Guided Biopsy** | | **Reference Standard of Truth Result** | | | | | | |
| --- | --- | --- | --- | --- | --- | --- | --- | --- |
|  |  | Tuberculosis | Non-tuberculosis Mycobacteria | Aspergillus | Coccidioidomycosis | Other Fungal | Other | Total |
| **Index Pathology Result** | Tuberculosis | 0 | 0 | 0 | 0 | 0 | 0 | 0 |
|  | Non-tuberculosis Mycobacteria | 0 | 0 | 0 | 0 | 0 | 0 | 0 |
|  | Aspergillus | 0 | 0 | 1 | 0 | 0 | 0 | 1 |
|  | Coccidiodomycosis | 0 | 0 | 0 | 0 | 0 | 0 | 2 |
|  | Other Fungal | 0 | 0 | 0 | 0 | 2 | 0 | 0 |
|  | Other | 0 | 0 | 0 | 0 | 0 | 7 | 7 |
|  | Total | 0 | 0 | 1 | 0 | 2 | 7 | 10 |
| **Robotic Computed Tomography Guided TransThoracic Needle Biopsy** | | **Reference Standard of Truth Result** | | | | | | |
|  |  | Tuberculosis | Non-tuberculosis Mycobacteria | Aspergillus | Coccidioidomycosis | Other Fungal | Other | Total |
| **Index Pathology Result** | Tuberculosis | 4 | 0 | 0 | 0 | 0 | 0 | 4 |
|  | Non-tuberculosis Mycobacteria | 0 | 5 | 0 | 0 | 0 | 0 | 5 |
|  | Aspergillus | 0 | 0 | 8 | 0 | 0 | 0 | 8 |
|  | Coccidiodomycosis | 0 | 0 | 0 | 5 | 0 | 0 | 5 |
|  | Other Fungal | 0 | 0 | 0 | 0 | 8 | 0 | 8 |
|  | Other | 0 | 0 | 0 | 0 | 0 | 8 | 8 |
|  | Total | 4 | 5 | 8 | 5 | 8 | 8 | 38 |

NSCLC: Non-small cell lung carcinoma; SCLC: Small cell lung carcinoma; CBCT-GB: Cone Beam Computed Tomography Guided Bronchoscopy; CT-TTNB: Computed Tomography Guided TransThoracic Needle Biopsy.

^a^This table shows into what category every index infection result was put into after 12 months of follow up data.

**OTHER TABLES AND FIGURES**

**e-Table 16. Diagnostic Accuracy and Diagnostic Yield Stratified by Tool-In-Lesion Confirmation**

| Outcome | CBCT-GB  (N = 392) | CT-TTNB  (N = 503) | *P*-Value | Odds Ratio  (95% CI)^a^ |
| --- | --- | --- | --- | --- |
| Primary Outcome – No./Total No. (%)^b^ |  |  |  |  |
| TIL Confirmed^c^ | 362/375 (96.5) | 494/503 (98.2) | 0.130 | 0.983 (0.961 – 1.005) |
| 24 month DA by TIL status |  |  |  |  |
| TIL Confirmed | 320/347 (92.2) | 434/468 (92.7) | 0.789 | 0.994 (0.956 – 1.035) |
| TIL Not Confirmed | 8/11 (72.7) | 6/7 (85.7) | 0.623 | 0.848 (0.529 – 1.360) |
| 12 month DA by TIL status |  |  |  |  |
| TIL Confirmed | 327/355 (92.1) | 436/471 (92.6) | 0.895 | 0.995 (0.956 – 1.035) |
| TIL Not Confirmed | 8/12 (66.7) | 7/8 (87.5) | 0.603 | 0.762 (0.472 – 1.229) |
| DY per ATS-ACCP by TIL status |  |  |  |  |
| TIL Confirmed | 307/362 (84.8) | 421/494 (85.2) | 0.923 | 0.995 (0.940 – 1.053) |
| TIL Not Confirmed | 7/13 (53.8) | 7/9 (77.8) | 0.380 | 0.692 (0.375 – 1.277) |
|  |  |  |  |  |

DA: Diagnostic Accuracy; DY: Diagnostic Yield; ATS: American Thoracic Society; ACCP: American College of Chest Physicians; CBCT-GB: Cone Beam Computed Tomography-Guided Biopsy; CT-TTNB: Computed Tomography Guided Transthoracic Needle Biopsy; TIL: Tool-In-Lesion; OR: Odds Ratio; CI: Confidence Interval.

^a^Shown are the OR of CBCT-GB Group relative to CT-TTNB Group giving the outcome of interest

^b^See Table 1 for varying definitions of diagnostic accuracy and diagnostic yield.

^c^TIL is confirmed when the biopsy tool is seen to be within the lesion on an intra-operative CT image.

**e-Figure 1: Flow Diagram showing convex EndoBronchial UltraSound use for lymph node biopsies and outcomes**

CBCT-GB: Cone Beam Computed Tomography-Guided Bronchoscopy; CT-TTNB: Computed Tomography-Guided TransThoracic Needle Biopsy; cEBUS: Convex EndoBronchial UltraSound; PPL: Peripheral Pulmonary Lesion; LN: Lymph Node.

^a^Patients suspected of having malignancy without extra-thoracic metastases who also have either a PPL in central two thirds of lung fields, PPL size >= 3cm, or have enlarged or FDG avid mediastinal or hilar lymph nodes^37, 38^.

**e-Figure 2: Statistical Analysis Flow Diagram**

CBCT-GB: Cone Beam Computed Tomography-Guided Bronchoscopy; CT-TTNB: Computed Tomography Guided TransThoracic Needle Biopsy; DA: Diagnostic Accuracy; DY: Diagnostic Yield; ATS-ACCP: American Thoracic Society-American College of Chest Physicians; CI: Confidence Interval; OR: Odds Ratio; LTFU: Lost to follow up; RAB: Robotic-Assisted Bronchoscopy; EBUS: endobronchial ultrasound
